# Discrete Cu(i) complexes for azide–alkyne annulations of small molecules inside mammalian cells†
†Electronic supplementary information (ESI) available. See DOI: 10.1039/c7sc04643j


**DOI:** 10.1039/c7sc04643j

**Published:** 2018-01-15

**Authors:** Joan Miguel-Ávila, María Tomás-Gamasa, Andrea Olmos, Pedro J. Pérez, José L. Mascareñas

**Affiliations:** a Centro Singular de Investigación en Química Biolóxica e Materiais Moleculares (CIQUS) , Departamento de Química Orgánica , Universidade de Santiago de Compostela , 15782 Santiago de Compostela , Spain . Email: joseluis.mascarenas@usc.es; b Laboratorio de Catálisis Homogénea , Unidad Asociada al CSIC , CIQSO-Centro de Investigación en Química Sostenible , Departamento de Química , Universidad de Huelva , Campus de El Carmen s/n , 21007 Huelva , Spain . Email: Perez@dqcm.uhu.es

## Abstract

The archetype reaction of “click” chemistry, namely, the copper-promoted azide–alkyne cycloaddition (CuAAC), has found an impressive number of applications in biological chemistry. However, methods for promoting intermolecular annulations of exogenous, small azides and alkynes in the complex interior of mammalian cells, are essentially unknown. Herein we demonstrate that isolated, well-defined copper(i)–tris(triazolyl) complexes featuring designed ligands can readily enter mammalian cells and promote intracellular CuAAC annulations of small, freely diffusible molecules. In addition to simplifying protocols and avoiding the addition of “non-innocent” reductants, the use of these premade copper complexes leads to more efficient processes than with the alternative, *in situ* made copper species prepared from Cu(ii) sources, tris(triazole) ligands and sodium ascorbate. Under the reaction conditions, the well-defined copper complexes exhibit very good cell penetration properties, and do not present significant toxicities.

## Abstract 2

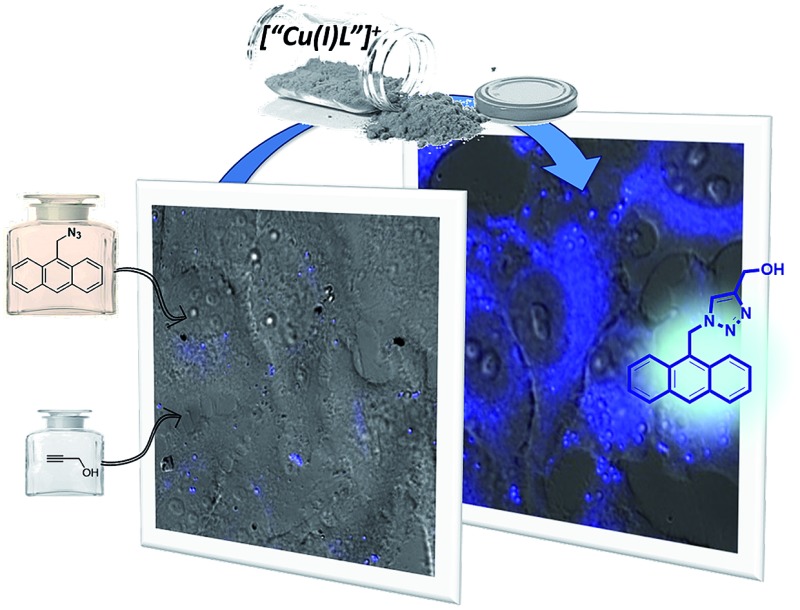
Cu(i) complexes do achieve azide–alkyne annulations of freely difusible small molecules inside mammalian cells.

## Introduction

Organometallic catalysis has changed the field of organic synthesis in the last half century, and has found important applications in other areas such as materials, energy or environmental sciences. In spite of such wide impact, the use of transition metal catalysis in biological contexts remains under-developed, probably due to the general belief that metal-promoted reactions are incompatible with the air atmospheres and aqueous environments of biological habitats, and that the metal complexes can be highly cytotoxic.^1^

**Fig. 1:**
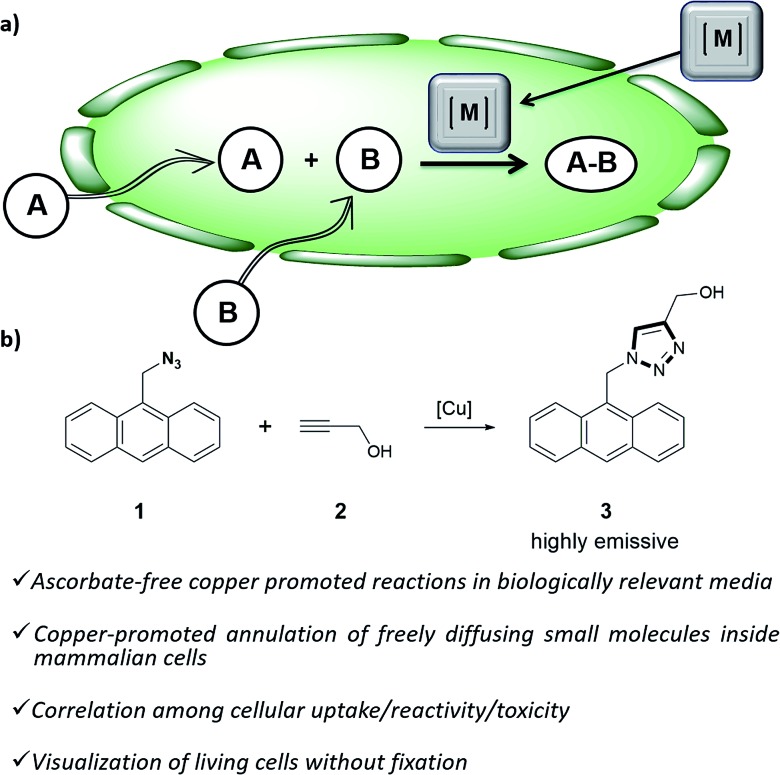
(a) Outline of a metal-promoted bimolecular coupling of exogenous molecules in living cells; (b) CuAAC reaction between anthracenyl azide **1** and propargyl alcohol **2**.

Only recently, a few examples demonstrating the viability of achieving transition metal promoted transformations in biological contexts,^2^ and even in intracellular environments,^3^ have been disclosed. Most of these reports deal with palladium or ruthenium-catalyzed uncaging of designed substrates equipped with inactivating handles.^4^ More challenging intracellular metal-promoted coupling reactions involving two different abiotic precursors are much scarcer. This is not surprising, as these reactions require the cell entrance and “meeting” of up to three different partners, namely the metal complex and two exogenous reactants (Fig. 1a). Thus, while several groups have demonstrated the viability of achieving Suzuki or Sonogashira couplings on appropriately modified proteins in *E. coli*,^5^ the only two examples described so far in mammalian cells involve the use of palladium nanoparticles, and fixed cells.^6^

Curiously, the well-known copper(i) catalyzed azide–alkyne cycloaddition (CuAAC) reaction,^7^ has been very scarcely explored inside the complex environment of living mammalian cells. Thus, while early work on the use of this reaction for biological purposes was restricted to bacteria,^8^ most of the other “*in vivo*” applications have been limited to the modification of cell surface labelled glycans.^9^ Probably, the established notion that copper is highly cytotoxic, and the requirement of excess of several additives, including “non-innocent” ascorbate, have precluded further research to implement the reaction in the challenging and crowded atmosphere of mammalian cells.9d,^10^

In recent years, several groups have developed water soluble ligands for Cu(i) that accelerate the reaction and also act as sacrificial scavengers/reductants of the reactive oxygen species (ROS) generated by copper, decreasing its cellular toxicity.^11^ The accelerating effect can be further improved if the copper chelated ligand is covalently linked to the azide, a tactic that has even been used for the intracellular labelling of proteins. However, with this strategy, the copper complex is likely sequestered by the products, owing to the presence of the triazole and the copper chelating moiety.^12^

Water soluble copper ligands linked to a cell penetrating peptide have been recently used to promote click reactions inside cells, but with low efficiency, and only for alkyne-modified proteins.10*b* If the modification of intracellular proteins is a highly relevant, and far from trivial, goal, achieving intracellular copper promoted reactions between two “freely diffusing small molecules” is even more challenging. Acceding to this type of reactivity can open new, exciting opportunities for biological or metabolic intervention, and for a metal-dependent generation of active drugs or optical signals. To the best of our knowledge, the only example of such type of intracellular CuAAC reaction relies on the use of cross-linked copper containing polymers termed metalorganic nanoparticles (MONPs), and requires high concentration of sodium ascorbate.^13^ Another class of copper nanostructures that can also promote the reaction in water has been recently reported, however their activity is confined to the extracellular milieu.^14^

Herein we report the first examples of an intracellular CuAAC transformation involving two exogenous, freely spreading substrates (small molecule azide and alkyne), promoted by discrete Cu(i) complexes (Fig. 1b). We also present data on the compared reactivity, redox stability, cell uptake and toxicity of *in situ* made copper species *versus* Cu(i) predefined complexes. These studies allow for the discovery of an independently isolated, well-defined Cu(i) complex equipped with the BTTE ligand (3-4-{{bis{[1-(1,1-dimethylethyl)-1*H*-1,2,3-triazol-4-yl]methyl}amino}methyl}-1*H*-1,2,3-triazole-1-ethanol, **L3**), which performs much better than the *in situ* mixture obtained from the ligand, a Cu(ii) source and sodium ascorbate.

## Results and discussion

**Chart 1:**
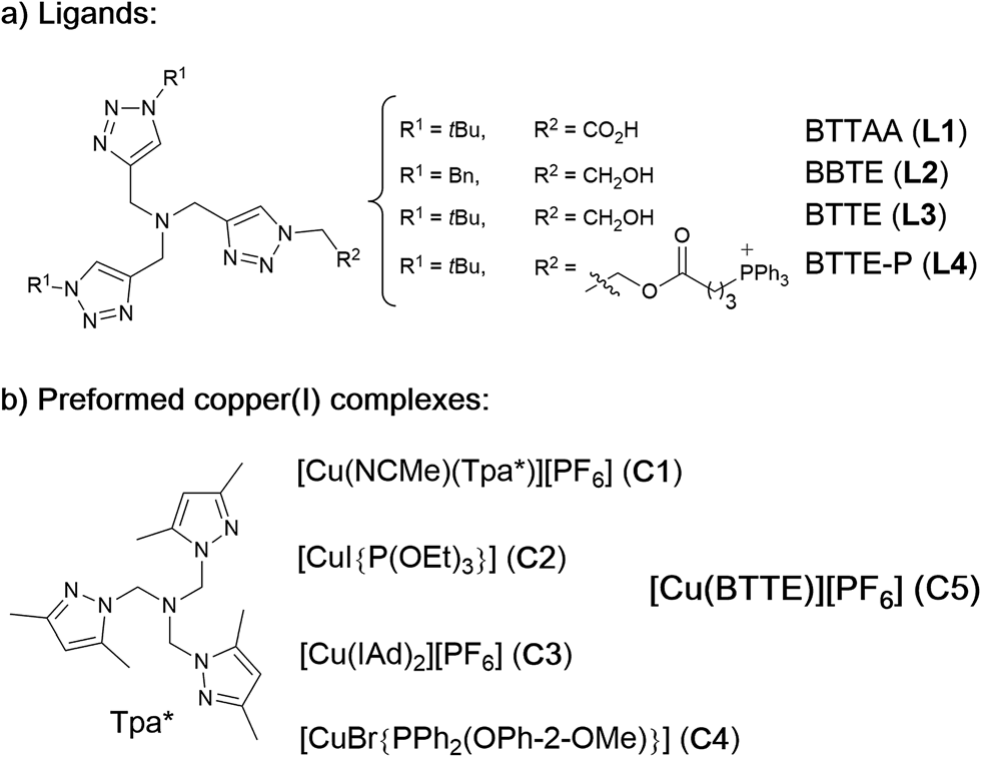
(a) Structure of water soluble tris(triazolylmethyl)amine ligands; (b) preformed, isolated Cu(i) complexes.

Our work was conceived on the hypothesis that designed, well-defined Cu(i) complexes might cross cell membranes and keep their oxidation +1 state under the reductive atmosphere of the cell. Thus we proposed to study the intracellular reactivity of tris-triazolyl–Cu(i) complexes generated *in situ* by reduction of Cu(ii) precursors with ascorbate, as well as of isolated, well-defined Cu(i) complexes (Chart 1).^15^ As substrates we chose fluorogenic azides that undergo an increase in fluorescent emission upon annulation with the corresponding alkynes.^16^ The most habitual azide substrate for these purposes is 3-azido-7-hydroxycoumarin, however, in our hands, preliminary control tests with HeLa cells indicated that this azide presents a substantial background signal. We therefore moved to the 9-(azidomethyl)anthracene (**1**, Fig. 1b) which is almost non-fluorescent, but undergoes a *ca.* 150-fold increase in fluorescence upon its annulation with alkynes (Fig. S2†). This increment can be explained in terms of suppression of the internal PET (photoinduced electron transfer) quenching on moving from the azide to the triazole structure.^16^,^17^

As tris(triazolylmethyl)amine ligands we selected BTTAA (3-4-{{bis{[1-(1,1-dimethylethyl)-1*H*-1,2,3-triazol-4-yl]methyl}amino}methyl}-1*H*-1,2,3-triazole-acetic acid, **L1**), which has been shown to be rather effective in CuAAC in aqueous media, and even in *E. coli*.9*c* We also prepared the analogues BBTE (**L2**) and BTTE (**L3**), which feature a hydroxyl group susceptible of conjugation to different units. Indeed, we synthesized the derivative **L4** which contains a triphenylphosphonium moiety designed to favor cellular internalizations and, eventually, mitochondrial localizations (Chart 1a). As predefined Cu(i) catalysts, we initially aimed to explore several previously characterized species such as pyrazolyl, NHC (N-heterocyclic carbene) phosphite or phosphinite copper complexes (**C1–C4**, Chart 1b). It is surprising that the catalytic activity of this type of well-defined Cu(i) complexes had never been explored in bio-relevant settings.

Before moving to cellular environments, we investigated the performance of the above complexes in aqueous media. With ligands **L1–L4**, the catalytic reactions were carried out using 75 mol% of copper, by mixing CuSO_4_ with 2 equiv. of the ligand in water (with 2% DMSO) at room temperature for 10 min, and adding the solution to either water or PBS (phosphate buffered solution) mixtures of anthracenyl azide **1** (100 μM) and propargyl alcohol **2** (200 μM), followed by sodium ascorbate (NaAsc, over 30 equiv.).

**Fig. 2:**
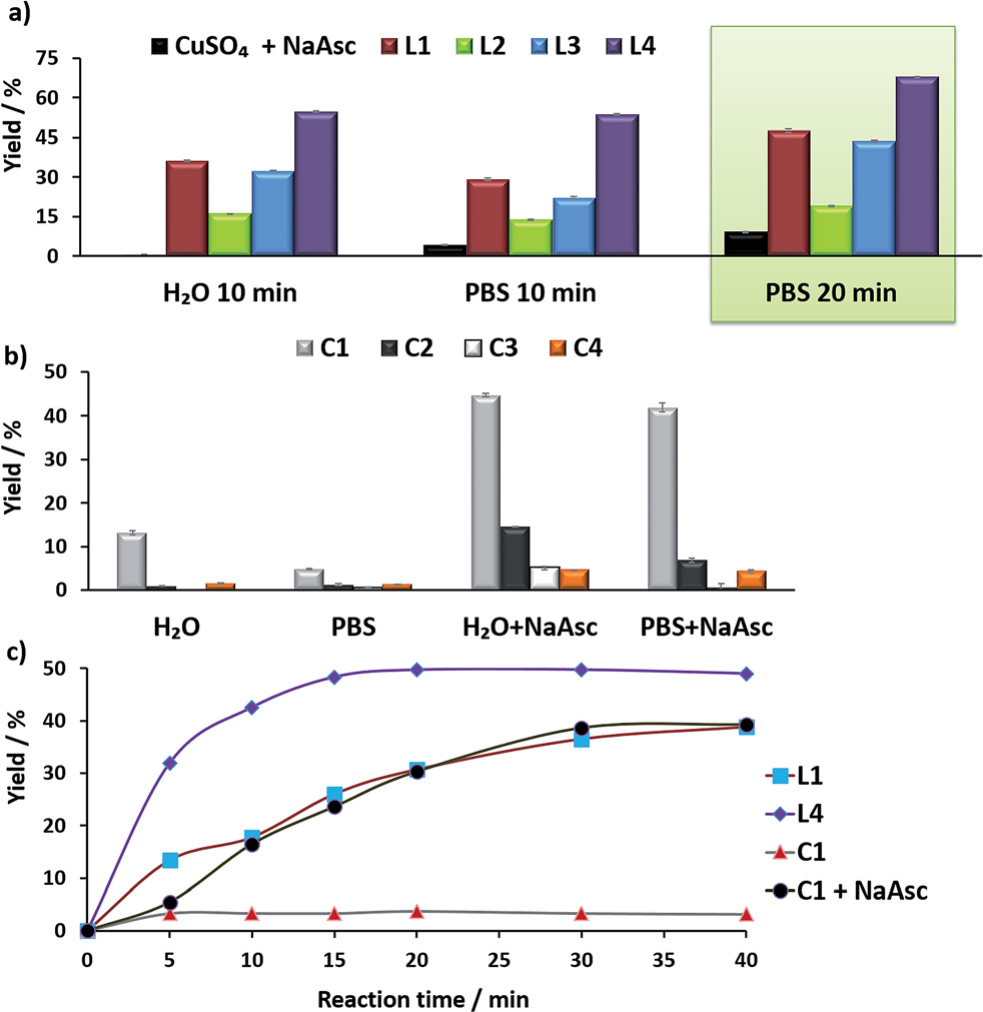
(a) Yields of the CuAAC with *in situ* preformed copper complexes. Reaction conditions: CuSO_4_ (75 μM) was mixed with 2 equiv. of the ligands **L1–L4** in H_2_O and the mixture was added to another solution containing anthracenyl azide **1** (100 μM) and propargyl alcohol **2** (200 μM) in either H_2_O or PBS. Then, NaAsc was added (2.5 mM) and the reaction was maintained at 25 °C; (b) yields of the CuAAC with the preformed copper complexes **C1–C4** (75 μM), using the above concentrations of reactants, with/without sodium ascorbate (2.5 mM), at 37 °C, 20 min; (c) conversion profiles of the CuAAC with different Cu(i) complexes in PBS, in reactions carried out with 25 μM of the copper species. The reaction yields were calculated using a fluorescence calibration curve that was obtained with increasing concentrations of the triazole **3**, from 0 to 100 μM.

For comparison purposes, we analyzed the conversion after 10 and 20 min, by using calibration curves (see Fig. 2 and Section S4 in the ESI†).

In the absence of ligands, *i.e.*, when only CuSO_4_ and sodium ascorbate are employed, the reaction proceeds with poor yields (<10% even after 24 h, Fig. 2a, dark blue bars). However, with ligand **L3** the product was obtained in 32% yield in water and 22% in PBS, after 10 min, while **L2** was less effective. Notably, using the phosphonium containing ligand **L4** we observed 50% of the triazole after 10 min, in both water and PBS (phosphate-buffered saline, Fig. 2a, purple bars), and a very good 70% yield after 20 min. As expected, if we skip the pre-treatment of the Cu(ii) complexes with sodium ascorbate, there is no reaction. UV-Vis and ^1^H-NMR analysis confirmed that mixing CuSO_4_, the ligand and sodium ascorbate generates a tris(triazole) Cu(i) species (Fig. S9 and S10†).

The performance of the predefined, isolated Cu(i) complexes **C1–C4** (Chart 1b) was also assessed in the absence or presence of ascorbate, at 37 °C (20 min, Fig. 2b). The carbene complex **C3** is almost inactive, and the phosphite and phosphinite complexes **C2** and **C4** also led to very poor conversions (less than 2% of the product). With the complex [Cu(NCMe)(Tpa*)][PF_6_] (**C1**)15a,b the reaction was slightly more efficient (13% in water and 5% in PBS). Importantly, addition of sodium ascorbate allowed much better conversions, specially, with **C2** and **C3**. These results suggest that under the reaction conditions (open air flask), the Cu(i) species are readily oxidized, something that was further confirmed by EPR. Therefore, while **C1** and **C2** are stable in solid state, in DMSO they are very rapidly oxidized under air to give paramagnetic Cu(ii) species (Fig. S11 and S12†).

Overall, the best conversions were achieved with the *in situ* made copper complexes in presence of ligand **L4**. Indeed, using this ligand it was possible to obtain the product in a satisfactory 46% yield, after 20 min, using just 25 μM of the copper source (Fig. 2c).^18^

With the above information in hand, we moved to living mammalian cells using two different cell lines: HeLa and A549 (living human cervical cancer cells and adenocarcinomic human alveolar basal epithelial cells, respectively). In the experiments with sodium ascorbate, the copper containing mixture added to the cells was prepared by mixing CuSO_4_ and the ligand (**L**) in a 1 : 2 ratio in water for 1 h, followed by treatment with an aqueous solution of sodium ascorbate (6 equiv.) for 30 min.^19^ With the defined, discrete Cu(i) species **C1–C4**, cells were directly incubated with a freshly made DMSO solution of the complexes. The experiments were carried out by mixing cultured cells with the copper solutions (75 μM for *in situ* made complexes and 50 μM for discrete Cu(i) species) for 30 min in fresh DMEM (Dulbecco's modified Eagle's medium), followed by two washing steps with DMEM prior to the addition of the reactants. The resulting cells were incubated with the azide **1** (100 μM) and the alkyne **2** (200 μM) in fresh DMEM for 60 min and washed twice with DMEM, before observation under the fluorescence microscope. It is important to note that we do not use cell fixation techniques, which allows for the preservation of the native living environment, and avoids artefacts or over-interpretations.

**Fig. 3:**
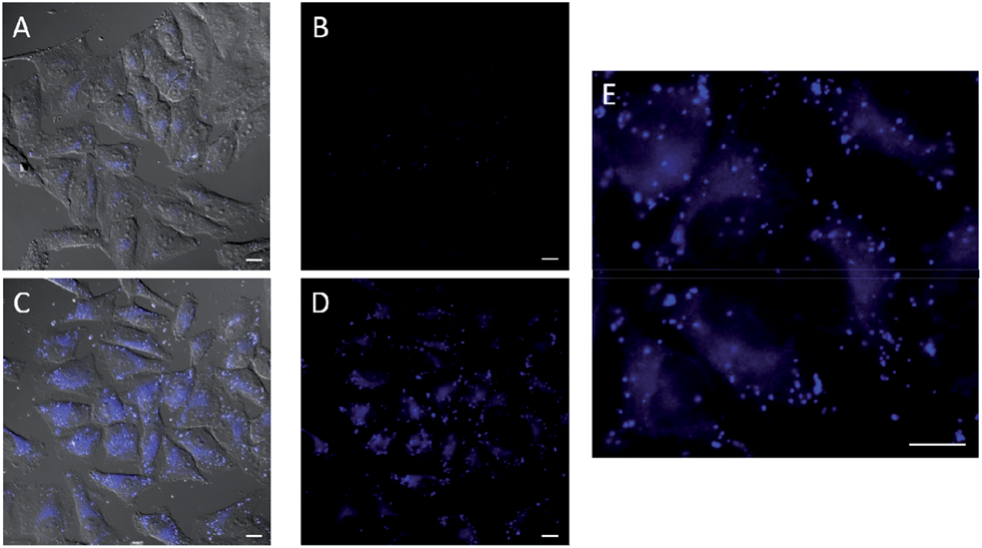
Fluorescence micrographies in experiments carried out in HeLa cells using the *in situ* made copper(i) complexes: 75 μM CuSO_4_, 2 equiv. **L4**, 6 equiv. NaAsc. (A, B) Cells incubated with azide **1** (100 μM) and alkyne **2** (200 μM) for 1 h, followed by double washing with DMEM (2 × 5 min). (C, D) Cells after incubation with the copper containing mixture (30 min), DMEM washings (2 × 5 min), and treatment with **1** (100 μM) and **2** (200 μM) for 1 h, followed by double washing with DMEM (2 × 5 min). (E) Zoom of panel D. Basal levels of fluorescence were normalized by LUT equalization. Scale bar, 12.5 mm. (A and C and brightfield).

In the experiments with *in situ* made copper species, in absence of ligands or with **L2**, we did not detect any intracellular fluorescence, while with **L1** and **L3** the fluorescent intensity was weak (Fig. S18†). However, we were glad to observe that when using **L4** as ligand, there was a clear blue intracellular fluorescence across the cytoplasm and in vesicles, with the cells showing an unaltered morphology (Fig. 3, panel C, D and E). Control experiments in absence of the copper species (Fig. 3, panel A and B), using the same threshold observation parameters, confirm that the signal must necessarily come from the expected reaction.^20^,^21^

Remarkably, despite their low *in vitro* activity, the predefined Cu(i) complex **C1**, the phosphite complex **C2** and the phosphinite complex **C4** were able to raise some intracellular fluorescence in experiments carried out in the absence of ascorbate, while **C3** failed to elicit any fluorescence (Fig. S19†).

Using MTT cytotoxicity assays we observed that more than 90% of the cells survived after 2 h of treatment with the standard Cu(ii)/**L4**/ascorbate mixture, using 75 μM of the copper source (80% survival after 12 h). With **C1** the cell survival was slightly lower, reaching values of approx. 80% after 2 h (Fig. S22†).

The reactivity observed with the tris(pyrazolyl) copper species **C1**, prompted us to pursue the specific preparation of a well-defined Cu(i) complex equipped with a tris(triazole) ligand. Thus, we focused on the isolation of a complex similar to **C1** but containing the ligand **L3** or **L4**. While with **L4** we have not yet been successful, we could isolate a Cu(i) complex (**C5**) by mixing [Cu(NCMe)_4_][PF_6_] with equimolar amounts of **L3** in methanol, and subsequent precipitation (Section S2†).

**Fig. 4:**
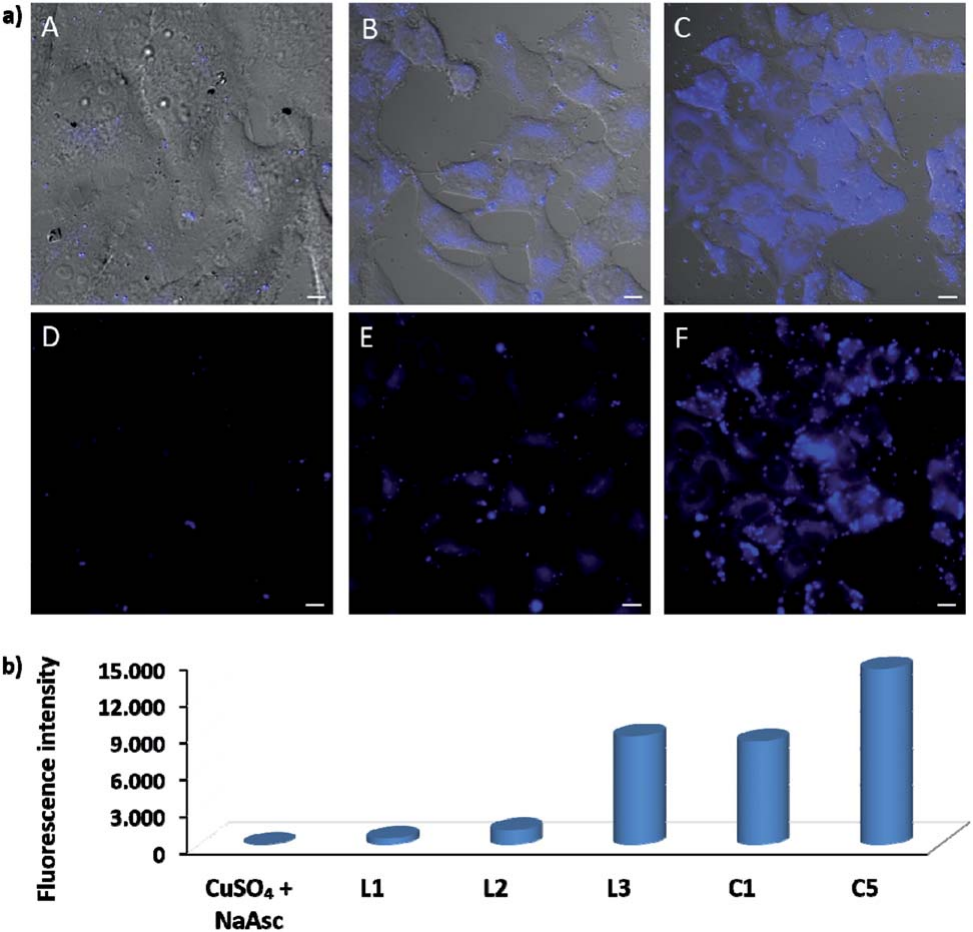
(a) Fluorescence micrographies resulting from the CuAAC reactions with the *in situ* made Cu(i)/**L3** species and with **C5**, using HeLa cells. (A, D) Cells incubated only with azide **1** (100 μM) and alkyne **2** (200 μM) for 1 h, followed by double washing with DMEM (2 × 5 min). (B, E) Cells after incubation with *in situ* made copper species with **L3**, using standard ascorbate reducing conditions (75 μM, 30 min incubation), DMEM washings (2 × 5 min), and treatment with **1** (100 μM) and **2** (200 μM) for 1 h, followed by double washing with DMEM (2 × 5 min). (C, F) Results using complex **C5**. A 10 mM solution of C5 in DMSO was freshly prepared in an open flask, and used immediately with no further precautions. Cells after incubation with **C5** (50 μM, 30 min incubation), DMEM washings (2 × 5 min), and treatment with **1** (100 μM) and **2** (200 μM) for 1 h, followed by double washing with DMEM (2 × 5 min). Basal levels of fluorescence were normalized by LUT equalization. Scale bar, 12.5 mm; (b) flow cytometry analysis for the quantification of fluorescent cells after the reactions promoted by copper complexes. The results with **L1–L3** refer to the copper-promoted reactions using these ligands and NaAsc (standard conditions). (A, B and C are brightfield).

EPR monitoring of fresh DMSO solutions of this complex (**C5**) demonstrated a higher redox stability than **C1**. Therefore, while in the case of **C1**, 80% of Cu(i) is oxidized to Cu(ii) after 20 min, under the same conditions, less than 30% of **C5** was oxidized (Fig. S12 and S13†). The *in vitro* performance of complex **C5** was quite similar to that of tris(pyrazolylmethane)-containing complex **C1**, however we were pleased to observe that this complex presents an excellent performance in native cellular settings, in the absence of sodium ascorbate (Fig. 4a, panel C and F); much better than that observed when the cells are incubated with the standard pre-made mixture containing Cu(ii)/**L3**/ascorbate (Fig. 4a, panel B and E).

To better appreciate the differences in efficiency, we have established a protocol to calculate reaction yields of the intracellular transformations, based on fluorescence measurements using a microplate reader (see Section S13 in the ESI†). The data were normalized with respect to the amount of anthracenyl azide (**1**, limiting reactant) uptaken by cells. Gratifyingly, when complex **C5** was used, the product was obtained in approx. 18% yield, which is over 7 times greater than that obtained using the *in situ* prepared complex with ligand **L3**.

The intracellular reactivity was also analyzed by flow cytometry, which confirmed that cells treated with **C5** presented higher levels of fluorescence when compared with that resulting from the *in situ* made **L3**/copper complex. Indeed, **C5** performed the best among all the copper species so far studied (Fig. 4b). The use of an extensive washing protocol to remove extracellular copper should assure that the reactions are taking place inside the cells. However, we further confirmed this by observing a total lack of reactivity in control experiments using extracellular media. Furthermore, we also observed that adding copper chelators like EDTA to the extracellular solution, in experiments carried out with living cells, has no effect on the results (see Fig. S20†).

Interestingly, there is a clear correlation between the copper uptake and the observed activity. Therefore, the phosphonium containing ligand **L4** promoted a relatively high intracellular accumulation of copper. The ICP-MS analysis also indicates that the well-defined Cu(i) complexes **C1** and **C2** are very well internalized, which explains why we do observe some intracellular reactivity despite their poor *in vitro* activity. More important, the copper complex **C5** is also very well internalized, leading to almost three times more internal copper than that from the corresponding *in situ* made copper complex with the ligand **L3**.

Therefore, the better intracellular performance of **C5***versus* the *in situ* made mixture of CuSO_4_/**L3**/NaAsc appears to be, at least in part, associated to an improved internalization.

**Fig. 5:**
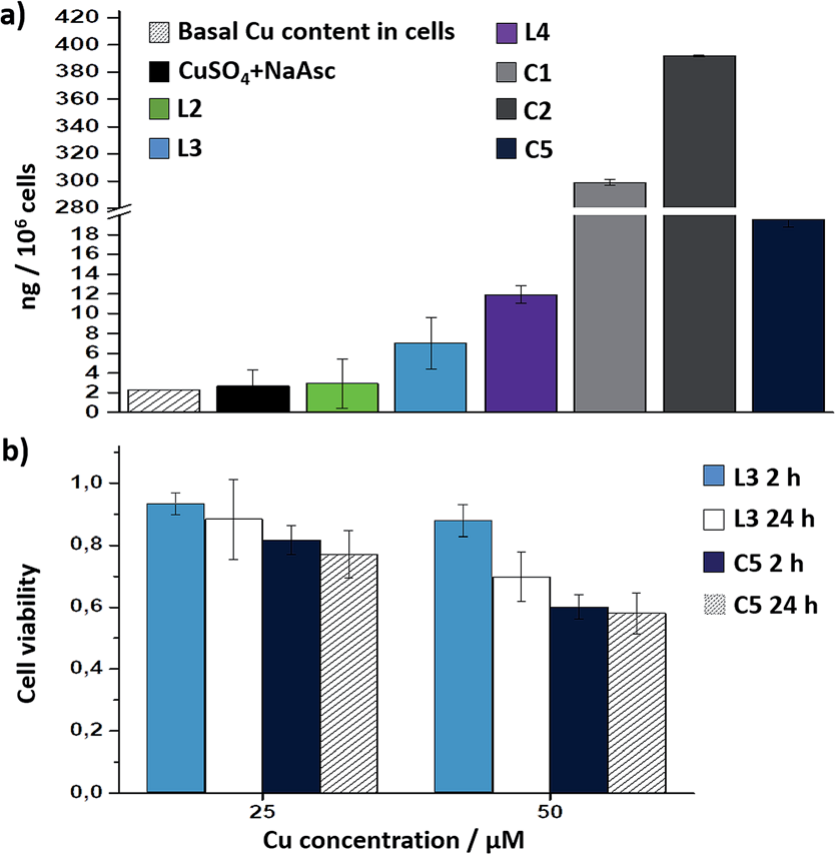
(a) ICP-MS results of the intracellular accumulation of copper after incubation of cells in DMEM with 75 μM of copper complexes (in DMSO) for 2 h, double washing with PBS (2 × 5 min) and digestion with HNO_3_. Note that when indicating **L2**, **L3** or **L4**, the results refer to the copper accumulation using these ligands and NaAsc (standard conditions); (b) viability assays with *in situ* made Cu(i) complexes with **L3**, and with preformed **C5** for 2 h and 24 h; the amount of viable cells was analysed by MTT assays.

Cell toxicity studies using different concentrations of **C5**, indicated over 70% viability after 2 h, which is raised to 82% using 25 instead of 50 μM (Fig. 5b and S22†). If we normalize these values with respect to the amount of copper internalized by cells (ICP data) we can conclude that the toxicity of complex with **L3**, and complex **C5** is similar. An additional control experiment indicated that the intracellular reaction is also feasible with 25 μM of **C5**, albeit the efficiency is slightly lower (over 9–10% yield, page S31).

The above information confirms that the effectivity of an intracellular CuACC with **C5** is associated to a good balance between redox stability and catalytic reactivity, and to its improved cell uptake properties, and furthermore confirms the viability of obtaining efficient, well-defined Cu(i) catalysts to be used in complex intracellular environments.

## Conclusions

We have demonstrated that water soluble copper(i) complexes featuring designed ligands can readily enter mammalian cells and promote intracellular CuAAC annulations of small, abiotic and freely diffusible molecules.

Our results indicate that using appropriate ligands, it is possible to tune the cell uptake and reactivity of Cu(i) complexes, and importantly, confirm the viability of using discrete copper species to promote efficient CuAAC annulations in the challenging interior of mammalian cells. Indeed, an independently isolated Cu(i)–tris(triazolylmethyl)amine complex, **C5**, that can be stored without degradation when kept under nitrogen, is capable of promoting the intracellular transformation even in the absence of ascorbate. This new complex displays better cellular uptake and better intracellular reactivity than that observed for the *in situ* made Cu(i)/**L3** complex.

This complex is therefore working as an “off-the-shelve” catalyst to promote challenging intermolecular annulations inside mammalian cells. The copper complex **C5** circumvents some of the actual limitations of the “*in vivo*” CuAAC chemistry, since it avoids the use of excess of ligands or the use of reductants such as ascorbate.

Current studies are focused on further improving the ligands to obtain even more effective catalysts that demonstrate negligible toxicity, on the development of copper complexes that can target different cellular organelles and on the use of the complexes for achieving designed biological alterations.

## Conflicts of interest

There are no conflicts to declare.

## Supplementary Material

Supplementary information
