# Supplementary material for: Discrete Cu(i) complexes for azide–alkyne annulations of small molecules inside mammalian cells
Source: Chem Sci. 2018 Jan 15;9(7):1947–52. doi: 10.1039/c7sc04643j (PMC5892125; doi:10.1039/c7sc04643j)
Supplement: Supplementary file 1 [file SC-009-C7SC04643J-s001.pdf]

Electronic Supplementary Material (ESI) for Chemical Science.
This journal is © The Royal Society of Chemistry 2018

ELECTRONIC SUPPLEMENTARY INFORMATION

Discrete Cu(I) Complexes for Azide-Alkyne Annulations of Small Molecules inside Mammalian Cells

Joan Miguel-Ávila,[a]‡ María Tomás-Gamasa,[a]‡ Andrea Olmos,[b] Pedro J. Pérez,* [b] and José L. Mascareñas*[a]

[a]Centro Singular de Investigación en Química Biolóxica e Materiais Moleculares (CIQUS) and Departamento de Química Orgánica, Universidade de Santiago de Compostela, 15782 Santiago de Compostela (Spain)

[b]Laboratorio de Catálisis Homogénea, Unidad Asociada al CSIC, CIQSO-Centro de Investigación en Química Sostenible, Departamento de Química, Universidad de Huelva Campus de El Carmen s/n, 21007 Huelva (Spain)

Table of Contents

S1. General information for chemical experiments
S2. Experimental procedures and data
S3. Spectroscopy studies on substrate 1 and product 3
S4. Catalytic CuAAC experiments under biological relevant conditions
    Selected in vitro experiments
S5. Quantification by HPLC-UV
S6. Spectroscopic analysis of the copper complexes
    UV-Vis, NMR and EPR experiments
S7. NMR spectra
S8. General information for biological experiments
S9. CuAAC experiments in living cells
S10. Cell viability
S11. ICP analysis
S12. Flow cytometry studies
S13. Quantification of the reaction inside living cells
S14. References

S1. General information for chemical experiments

Synthetic preparative procedures were performed under an atmosphere of dry nitrogen using vacuum-line and standard Schlenk techniques, unless otherwise indicated. The solvents for organic synthesis were of reagent grade unless otherwise noted. Dry solvents were directly purchased from Aldrich and used without further purification. *N*,*N*-dimethylformamide and trifluoroacetic acid were purchased from Scharlau. Water was deionized and purified on a *Millipore Milli-Q Integral* system.

Chemicals were purchased from *Sigma Aldrich*, *Alfa Aesar, Fluka* or *Acros Organics* and used without further purification.

The copper promoted catalytic reactions were carried out without particular precautions to extrude moisture or oxygen, and open to air, except when noted otherwise.

Chromatographic purification of products was accomplished using flash column chromatography on *Merck* Geduran Si 60 (40 – 63 μm) silica gel (normal phase) or by reversed-phase high-performance liquid chromatography (RP-HPLC). Thin layer chromatography (TLC) was performed on *Merck* 60 (silica gel F_{254}) plates and components were visualized by observation under UV, using staining solutions of $KMnO_4$ or *p*-anisaldehyde.

Concentration refers to the removal of volatile solvents via distillation using a rotary evaporator *Büchi R-210* equipped with a thermostated bath *B-491*, a vacuum regulator *V-850*, and a vacuum pump V-700, followed by residual solvent removal under high vacuum.

^{1}H NMR (300 MHz) spectra were recorded at room temperature on a *Varian Mercury* 300 MHz spectrometer and ^{1}H NMR (500 MHz) spectra were recorded at room temperature on a *Bruker DRX-500* spectrometer. ^{13}C NMR (125 MHz) spectra were recorded on a *Bruker DRX-500* spectrometer. ^{31}P NMR (202 MHz) spectra were recorded on a *Bruker DRX-500* spectrometer. The spectra were calibrated to the residual solvent peak, if possible. As an external reference triphenyl phosphate (-18 ppm) was used for ^{31}P NMR spectra. Multiplicities are abbreviated as follows: s = singlet, d = doublet, t = triplet, q = quartet, m = multiplet, br = broad and combinations of these.

NMR spectra were analyzed using *MestreNova© NMR* data processing software (www.mestrelab.com). The chemical shifts (δ) are given in ppm and the coupling constants (*J*) in Hz.

EPR measurements were performed with a *Bruker EMX* at a temperature of 120 K.

Fluorescence measurements were performed using a *Horiba FluoroMax®-3* Spectrophotometer with the following settings: increment 1.0 nm, averaging time 0.2 s, excitation slit width 2.0 nm, emission slit width 2.0 nm.

UV measurements were performed using a *Jasco V-670* spectrometer.

The evolution of the reactions was studied by fluorescence using a *Tecan infinite F200Pro* microplate reader, with a gain of excitation of 48%.

Electrospray Ionization Mass Spectrometry (ESI/MS) was performed with a *Bruker Amazon IT/MS* using direct injection of a solution of the compound into the MS.

Mass spectra were acquired using electrospray (ESI) and were recorded at the CACTUS facility of the University of Santiago de Compostela.

Analytical HPLC was performed on an *Agilent 1260 Infinity II* coupled to an *Agilent Technologies 6120 Quadrupole LC-MS* using a flow rate of 0.35 mL min^{-1} at room temperature. The initial conditions for the HPLC system were: H_2O/MeCN (50/50) followed by a gradual change over 35 min to H_2O/MeCN (0/100). The chromatogram was recoded via UV absorption at λ = 254 nm.

S2. Experimental procedures and data

9-(Azidomethyl)-anthracene (**1**),[1] 1-(9-anthracenylmethyl)-1*H*-1,2,3-triazole-4-methanol (**2**),[2] 3-4-{{bis{[1-(1,1-dimethylethyl)-1*H*-1,2,3-triazol-4-yl]methyl}amino}methyl}-1*H*-1,2,3-triazole-acetic acid (BTTAA **L1**), 4-{{bis{[1-(phenylmethyl)-1*H*-1,2,3-triazol-4-yl]methyl}amino}methyl}-1*H*-1,2,3-triazole-1-ethanol (BBTE **L2**), 3-4-{{bis{[1-(1,1-dimethylethyl)-1*H*-1,2,3-triazol-4-yl]methyl}amino}methyl}-1*H*-1,2,3-triazole-1-ethanol (BTTE **L3**),[4] (2-carboxiethyl)diphenyl(pyren-1-ylmethyl)phosphonium bromide,[5] [Cu{BrPPh$_2$(OPh-2-OMe)}] (**C4**),[6] bis(1,3-di(adamantan-1-yl)-1,3-dihydro-2H-imidazol-2-ylidene)copper ([Cu(IAd)$_2$][PF$_6$]) (**C3**)[7] and N-[(tris(3,5-dimethyl-1-*H*-pyrazol-1-yl)methyl))] [Cu(NCMe)(Tpa*)][PF$_6$] (**C1**)[8] were synthesized according to literature procedures and spectroscopic data are in agreement with the bibliographic ones.

2.1 Synthesis of ligands L1-L3

BTTAA (**L1**), BBTE (**L2**), and BTTE (**L3**) were prepared following the synthetic route shown in Scheme S1.

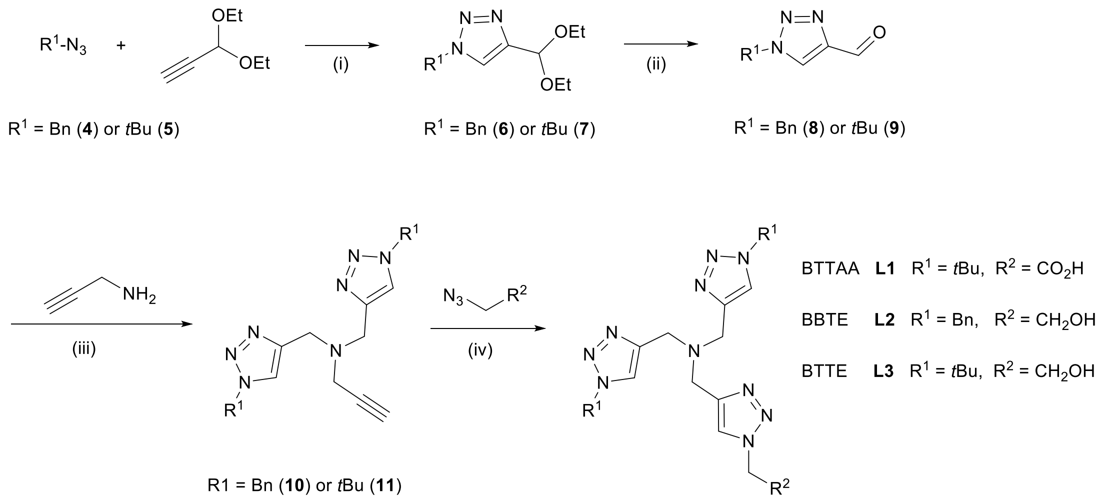

Scheme S1. Synthesis of the ligands **L1-L3**. Reagents and conditions: (i) **4** or **5** (1.15 equiv), 3,3-diethoxyprop-1-yne (1.0 eq), H_2O-*t*BuOH (1:1), $NaHCO_3$ (1.4 equiv), $CuSO_4{\cdot}5H_2O$ (5 mol%), sodium ascorbate (20 mol%), 12 h, room temperature, 95%; (ii) **6** or **7** (1.0 equiv), trifluoroacetic acid (2.3 equiv), dichloromethane, under nitrogen atmosphere, 5 h, room temperature, 85%; (iii) **8** or **9** (2.2 equiv), prop-2-yn-1-amine (1.0 equiv), Na(AcO)$_3$BH (2.5 equiv), 1,2-dichloroethane, 40 h, room temperature, 84%; (iv) **10** or **11** (1.0 equiv), 2-azidoetanol (1.5 equiv), $CuSO_4{\cdot}5H_2O$ (5 mol%), sodium ascorbate (20 mol%), 24 h, room temperature, 90%.

BBTE (L2):

^{1}H-NMR ($CDCl_3$, 300 MHz): δ (ppm) = 7.80 (s, 1H, C*H*=C), 7.62 (s, 2H, 2xC*H*=C), 7.36–7.28 (m, 6H, 6xArH), 7.23 (dd, 4H, 4xArH), 5.49 (s, 4 H, 2xAr-C*H*$_2$), 4.45 (t, 2H, N-CH$_2$-C*H*$_2$-OH), 4.01 (t, 2H, N-*CH*$_2$-CH$_2$-OH), 3.72 (s, 6H, 3xC*H*$_2$-N).

^{13}C NMR ($CDCl_3$, 126 MHz): δ (ppm) = 144.3 (2xN-CH=C), 143.7 (N-CH=*C*), 134.7 (ArC), 129.1 (ArC), 128.7 (ArC), 128.0 (ArC), 124.8 (N-*C*H=C), 123.8 (2xN-*C*H=C), 60.9 (N-CH$_2$-*C*H$_2$-OH), 54.1 (2xAr-*C*H$_2$), 52.7 (N-*C*H$_2$-CH$_2$-OH), 47.5 (N-*C*H$_2$), 46.9 (2xN-*C*H$_2$).

HRMS: Calculated for: $C_{25}H_{29}N_{10}O$: 485.2448; found: 485.2510.

2.2 Synthesis of ligand L4

{4-{2-{4-{{bis{[1-(*tert*-Butyl)-1H-1,2,3-triazol-4-yl]methyl}amino}methyl}-1H-1,2,3-triazol-1-yl}ethoxy}-4-oxobutyl}triphenylphosphonium bromide (BTTE-P L4)

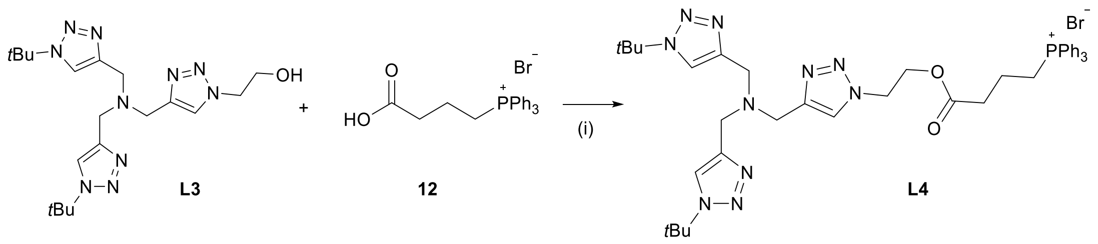

Scheme S2. Synthesis of the ligand L4. Reagents and abbreviations: (i) L3 (1.0 equiv), (3-carboxypropyl)triphenylphosphonium bromide **12** (1.0 equiv), EDC (2.5 equiv), DMAP (2.0 equiv), *N,N*-dimethylformamide, 12 h, room temperature, 55%. EDC = 1-Ethyl-3-(3-dimethylaminopropyl)carbodiimide; DMAP = 4-Dimethylaminopyridine.

(3-Carboxypropyl)triphenylphosphonium bromide **12** (94.0 mg, 0.22 mmol, 1.0 equiv) was dissolved in *N,N*-dimethylformamide (2 mL). 1-Ethyl-3-(3-dimethylaminopropyl)carbodiimide (EDC) (104.0 mg, 0.54 mmol, 2.45 equiv), BTTE (**L3**) (91.6 mg, 0.22 mmol, 1.0 equiv) and 4-dimethylaminopyridine (52.0 mg, 0.44 mmol, 2.0 equiv) were subsequivuently added to the solution. The reaction mixture was left stirring overnight and the solvent was removed under reduced pressure. The crude was purified by flash chromatography on silica gel using DCM / MeOH 9.5:0.5 as eluent. The product **L4** was obtained as a white solid (102 mg, 55%).

R$_f$ = 0.3 (DCM / MeOH 9:1) staining $KMnO_4$.

^{1}H NMR ($CDCl_3$, 500 MHz): δ (ppm) = 8.11 (s, 1H, N-C*H*=C), 7.89 (s, 2H, 2xN-C*H*=C), 7.81-7.71 (m, 9H, 9xArH), 7.64 (td, 6H, ^3J = 7.9, ^4J = 3.5 Hz, 6xArH), 4.65 (t, 2H, ^3J = 5.2 Hz, N-C*H*$_2$-CH$_2$-OH), 4.46 (t, 2H, ^3J = 5.2 Hz, N-C*H*$_2$-CH$_2$-OH), 3.81 (ddd, 2H, ^2J = 16.5, ^3J = 8.6, ^4J = 4.9 Hz, P-C*H*$_2$-CH$_2$), 3.70 (s, 2H, N-C*H*$_2$), 3.67 (s, 4H, 2xN-C*H*$_2$), 2.82 (t, 2H, ^3J = 6.3 Hz, CH$_2$-C*H*$_2$-CO), 1.90–1.77 (m, 2H, CH$_2$-C*H*$_2$-CH$_2$), 1.60 (s, 18H, 6xCH$_3$).

^{13}C NMR ($CDCl_3$, 126 MHz): δ (ppm) = 172.2 (CO), 144.0 (N-CH$_2$=*C*), 143.3 (2xN-CH$_2$=*C*), 135.1 (d, ^4J = 3.1 Hz, ArC), 133.6 (d, ^2J = 10.6 Hz, ArC), 130.5 (d, ^3J = 13.1 Hz, ArC), 125.1 (N-*C*H=C), 121.1 (2xN-*C*H=C), 118.0 (d, *J* = 86.3 Hz, ArC), 62.8 (N-CH$_2$-CH$_2$-OH), 59.2 (2x*C*-CH$_3$), 49.0 (N-*C*H$_2$-CH$_2$-OH), 47.6 (N-*C*H$_2$), 47.2 (2xN-*C*H$_2$), 33.1 (d, ^2J = 18.8 Hz, CH$_2$-CH$_2$-CH$_2$), 30.0 (6x*C*H$_3$), 21.5 (d, *J* = 52.0 Hz, CH$_2$-CH$_2$-P), 17.9 (d, ^3J = 2.5 Hz, CH$_2$-CH$_2$-CO).

^{31}P NMR ($CDCl_3$, 202 MHz): δ (ppm) = 22.6.

HRMS: Calculated for $C_{41}H_{52}N_{10}O_2P^+$: 747.4007; found: 747.4007.

2.3 Synthesis of complex Cu-BTTE PF_6 (C5)

A solution of the ligand **L3** (246 mg, 0.59 mmol, 1.0 equiv) in 15 mL of methanol was added via cannula over a solution of tetrakis(acetonitrile)copper(I) hexafluorophosphate (220 mg, 0.59, 1.0 equiv) in 25 mL of methanol. After a few minutes, formation of white precipitate was observed. The mixture was stirred for two hours at room temperature. Methanol was filtered off and the solid was washed with 10 mL of methanol and 10 mL of diethyl ether. The solid was dried under vacuum. Cu-BTTE PF_6 (**C5)** was isolated as a white solid (260 mg, 71%).

^{1}H NMR (DMSO-d$_6$, 500 MHz): δ (ppm) = 8.34 (s, 2H, 2xN-C*H*=C), 8.16 (s, 1H, N-C*H*=C), 5.07 (t, 1H, ^3J = 5.3 Hz, OH), 4.42 (t, 2H, ^3J = 4.9 Hz, N-CH$_2$-C*H*$_2$-OH), 3.85 (brs, 6H, 3xN-C*H*$_2$), 3.78 (brs, 2H, N-C*H*$_2$-CH$_2$-OH), 1.60 (s, 18H, 6xCH$_3$).

[13]**C NMR** (DMSO-d6, 125 MHz): δ (ppm) = 144.0 (2xN-CH=C), 124.7 (N-CH=C), 121.5 (3xN-CH=C), 60.3 (N-*C*-CH3), 59.5 (3xN-CH2), 53.0 (N-CH2-CH2-OH), 47.0 (N-CH2-CH2-OH), 29.2 (6xCH3).

Elemental analysis: Calc. ($C_{19}H_{32}CuF_6N_{10}OP$) 36.51 C, 5.16 H, 22.41 N; found: 36.86 C, 5.15 H, 22.12 N.

S3. Spectroscopy studies on substrate 1 and product 3

For the UV-Vis spectroscopic studies, solutions of the substrates **1** and **3** (5 mM in DMSO) were freshly prepared and diluted with 2 mL H_2O into a quartz Hellma® fluorescence cuvette with a pathlength 10x10 mm, chamber volume 3.5 mL (final concentration: 100 μM).

The samples were analyzed in a *Jasco V-630*UV-Vis spectrophotometer at 25 °C. UV-Vis analyses were performed in the interval of 300-800 nm.

For the fluorescence measurements, solutions of the compounds **1** and **3** (5 mM in DMSO) were freshly prepared and diluted with water into quartz Hellma® fluorescence cuvette with a path length 10x10 mm, chamber volume 3.5 mL (final concentration: 20 μM).

The samples were analyzed in a *Hoiba FluoroMax-3 fl*uorescence Spectrophotometer at 25 °C. The solutions of substrates were excited at 367 nm and the emission spectrum was recorded in the interval 355-700 nm.

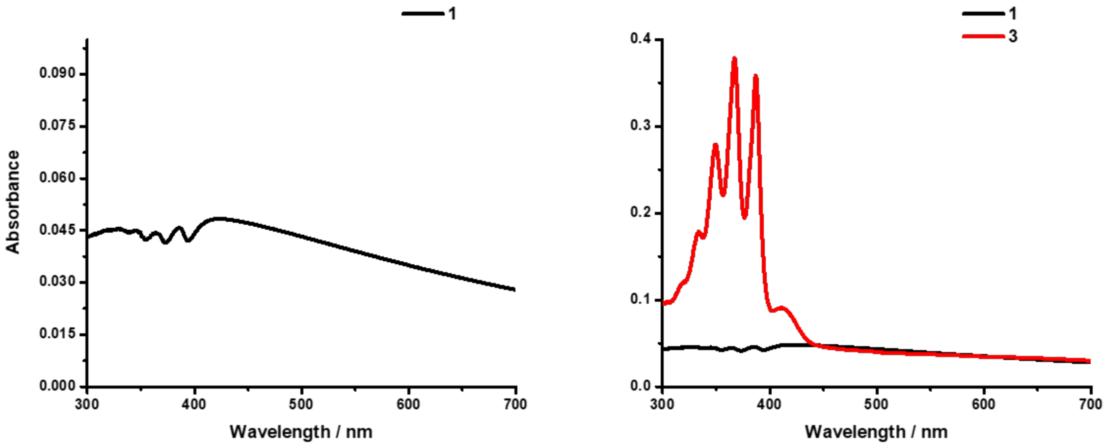

Figure S1. Comparison of UV/Vis absorption spectra of **1** (100 μM, black) and **3** (100 μM, red) in H_2O (2 mL).

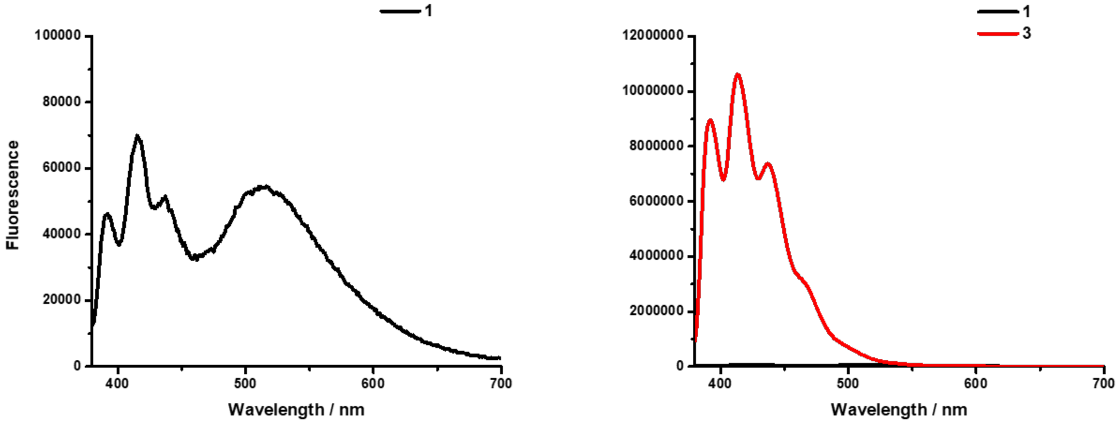

Figure S2. Comparison of fluorescence spectra of **1** (20 μM, black, no emission) and **3** (20 μM, red, λ_{em}= 515 nm) in H_2O (2 mL).

S4. Catalytic CuAAC experiments under biological relevant conditions

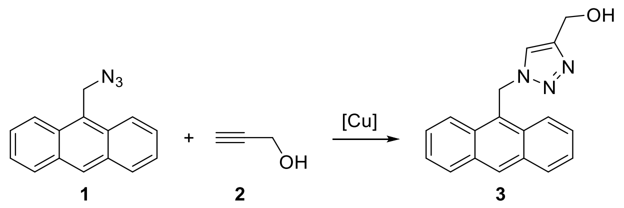

Scheme S3. CuAAC reaction between anthracenyl azide **1** and propargyl alcohol **2**.

The studies on the catalytic copper azide-alkyne cycloaddition of 9-(azidomethyl)-anthracene **1** and propargyl alcohol **2** under biologically relevant conditions were performed in a microplate of 96 wells.

Reaction with *in situ* prepared Cu(I) complexes using L1-L4 ligands, and with preformed Cu(I) complexes C1-C5

A fresh solution of $CuSO_4$ (12.5 µL, 10 mM in H_2O) was added to a fresh solution of the ligand (12.5 µL, 20 mM in H_2O and 5% DMSO, 2.0 equiv) in an eppendorf tube of 500 µL containing 25 µL of milliQ water (with **L2**, solubilization required a little heating). The mixture was stirred for 10 min at room temperature.

A stock mixture containing the reactants was prepared in a eppendorf tube by mixing a fresh solution of azide **1** (3.91 µL, 50 mM, DMSO) and a fresh solution of alkyne **2** (7.82 µL, 50 mM, H_2O), in 1688.27 µL of the corresponding solvent (water or PBS).

87 µL of this mixture was poured into the reaction well of the plate, and mixed with the Cu(II) containing solution (3 µL, 2.5 mM, 0.75 equiv). Finally, sodium ascorbate was added (10 µL, 25 mM, H_2O) to start the reaction, which was carried out under air at room temperature.

In case of the preformed Cu(I) complexes, a fresh solution of the complexes **C1**-**C5** (2.5 mM in DMSO) was freshly prepared. Then, 3 µL of this solution was added to the plate well containing the mixture with both substrates (87 µL) and diluted in the chosen solvent (3 µL of water or PBS, or the sodium ascorbate water solution). The experiments were carried out at 25ºC or 37ºC.

Final concentrations in the reaction mixtures: 100 µM azide **1**, 200 µM alkyne **2**, 75 µM copper, 2.5 mM sodium ascorbate (when present).

The results were analyzed by fluorescence measurements in a microplate reader (*Tecan infinite F200Pro*). Fluorescence data were obtained by using an excitation wavelength of 360 nm and recorded at 465 nm. Every measured value is the mean value of three aliquots.

<u>Calibration</u> experiments were performed in a 96 wells plate by addition of increasing quantities of a fresh solution of 1-(9-anthracenylmethyl)-1*H*-1,2,3-triazole-4-methanol **3** (1 mM or 5mM in DMSO) in milliQ water. Higher concentration results in the precipitation of the triazole **3**. The experiments were performed by triplicate, and measured in the fluorescence microplate reader (excitation: 360 nm, emission: 465 nm). The fluorescence results were processed with OriginPro 2016 to obtain the calibration equation and applied to quantify the fluorescence of the in vitro experiments.

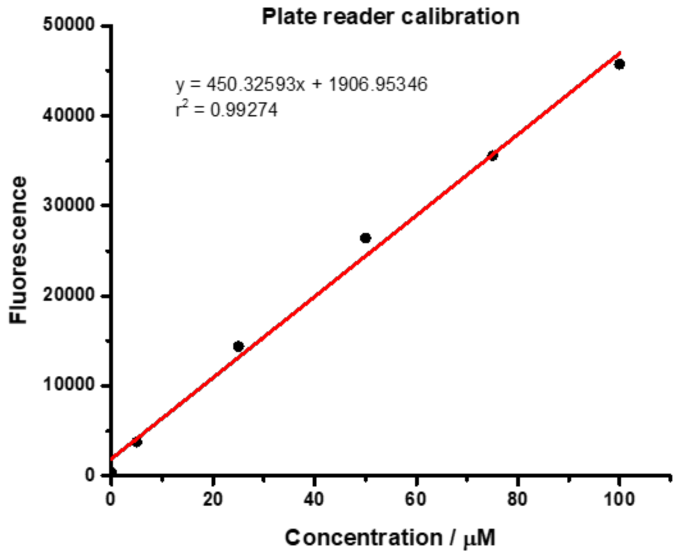

Figure S3. Standard curve of triazole **3**. Fluorescence intensity (λ_{ex} = 360 nm, λ_{em} = 465 nm) at different concentrations. Linear regression curve for 0–100 µM is shown.

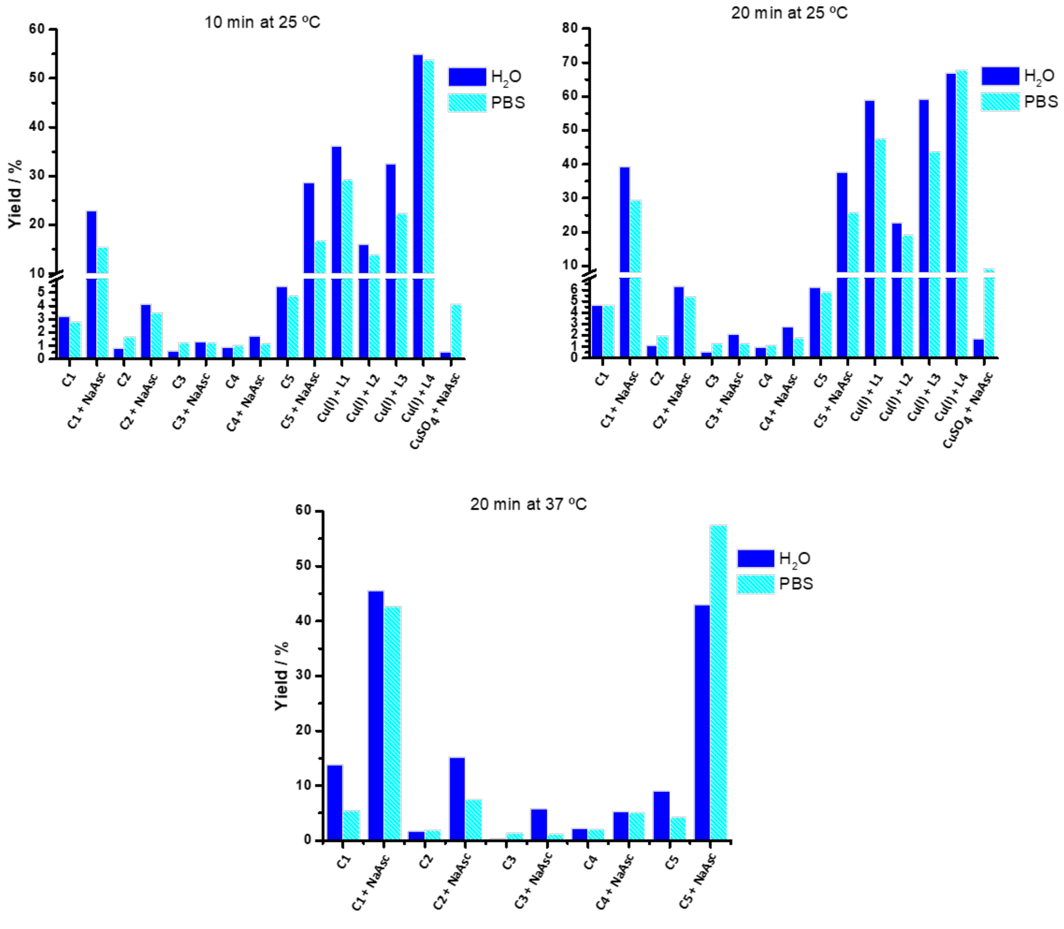

Figure S4. Summary of the results obtained for the CuAAC carried out in vitro at the indicated T[a]. **Cu(I) + Ln** refers to the in situ preformed complex with selected ligand **Ln**, $CuSO_4$ and NaAsc.

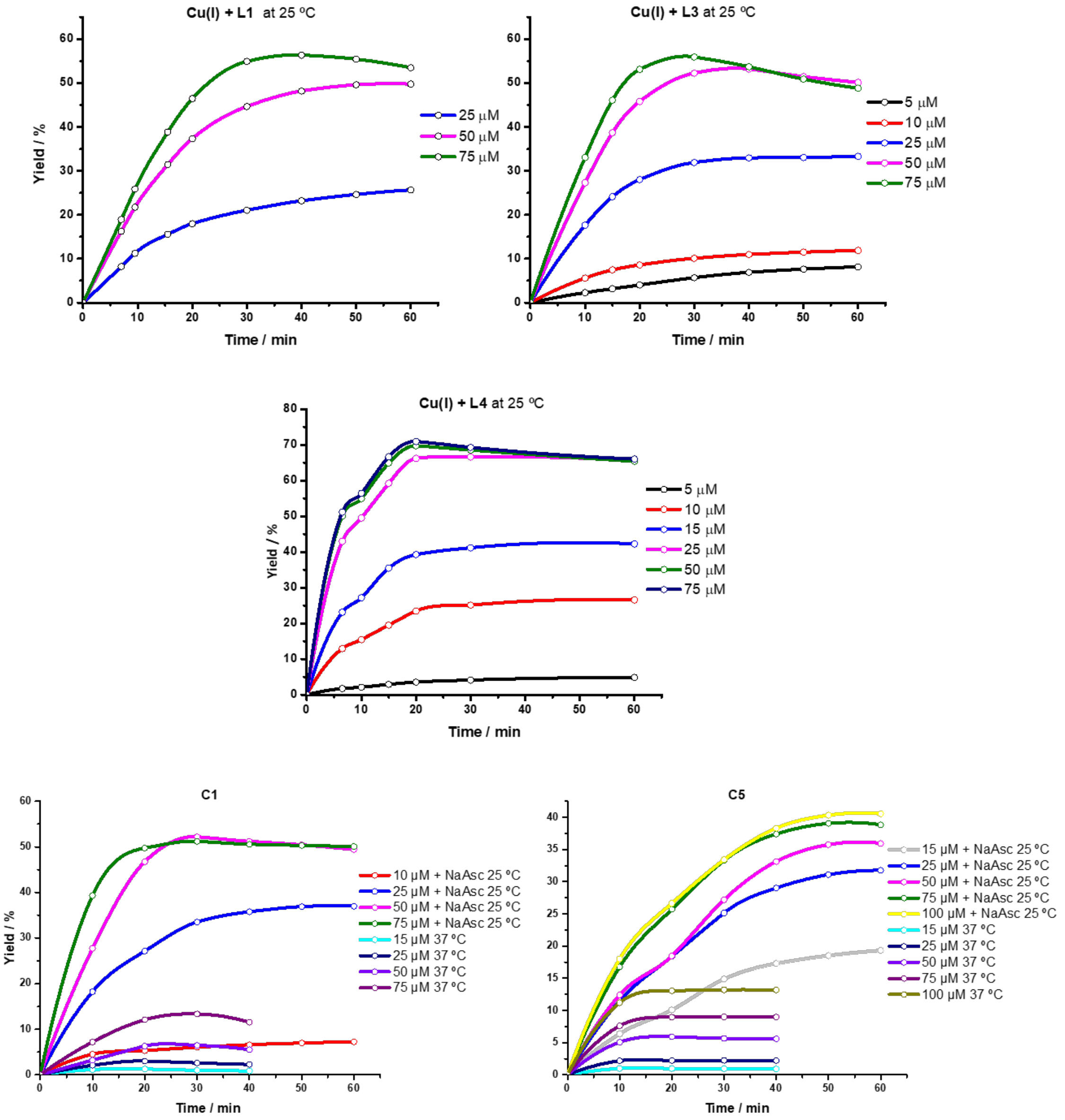

Figure S5. Selected in vitro experiments in water using different concentration of in situ preformed copper complexes with **L1**, **L3** and **L4,** and defined Cu(I) complexes **C1** and **C5**. The in situ prepared complex with **L3** allows comparison with complex **C5**. **Cu(I) + Ln** refers to the in situ preformed complex with selected ligand **Ln**, $CuSO_4$ and NaAsc.

S5. Quantification by HPLC-UV

General Procedure: The CuAAC reaction of **1** and **2** with the different complexes was also quantified by using analytical HPLC-UV methods.

The Cu(II) mixtures with the corresponding politriazol ligands were prepared by addition of fresh solutions of $CuSO_4$ (12.5 μL, 10 mM in H_2O) to solutions of the ligand (12.5 μL, 20 mM in H_2O and 5% DMSO) in an eppendorf tube of 500 μL containing 25 μL of milliQ water. The mixture was stirred for 10 min at room temperature under air.

The stock mixture containing the reactants was prepared in an eppendorf tube containing 1688.27 μL of water, by mixing a fresh solution of the azide **1** (3.91 μL, 50 mM, DMSO, 1 equiv) and a fresh solution of alkyne **2** (7.82 μL, 50 mM, H_2O, 2 equiv). The mixture was stirred for homogenization.

The copper containing mixture (15 μL, 2.5 mM, H_2O) was added to a eppendorf tube containing the above mixture with both reactants (435 μL). Finally, sodium ascorbate was added (50 μL, 25 mM, H_2O, 33 equiv), and the reaction carried under air at room temperature.

In case of the predefined Cu(I) complexes, a fresh solution of the complexes **C1**-**C5** (2.5 mM in DMSO) was freshly prepared and immediately added (15 μL, 2.5 mM, DMSO) to an eppendorf tube containing the above prepared solution with both substrates (435 μL).

Final concentrations: 100 μM azide **1**, 200 μM alkyne **2**, 75 μM copper complex and 2.5 mM sodium ascorbate (when present). The reaction was carried out under air and without stirring, unless otherwise is mentioned.

The formation of the products was confirmed by analytical HPLC-UV (254 nm), and quantified using a calibration curve with an internal standard. HPLC method: H_2O/MeCN gradient 95/5 ⟶ 5/95 in 12 min, then 3 min 100% MeCN.

Thecalibration of azide **1** and triazole **3** was performed in the same HPLC vial, using MeCN as main solvent. The different samples were prepared from a 5 mM fresh stock solution of the analyte in DMSO. Coumarine (870 μM as final concentration) was used as internal standard. The areas obtained from the chromatogram were processed with OriginPro 2016 to obtain the calibration equation and applied to quantify the absorbance of the in vitro experiments.

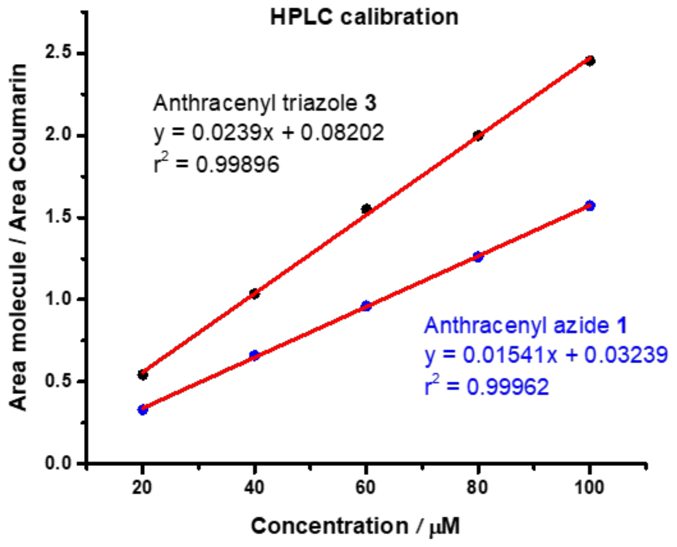

Figure S6. Standard curve of azide **1** and triazole product **3**. Linear regression curve for 0-100 μM is shown.

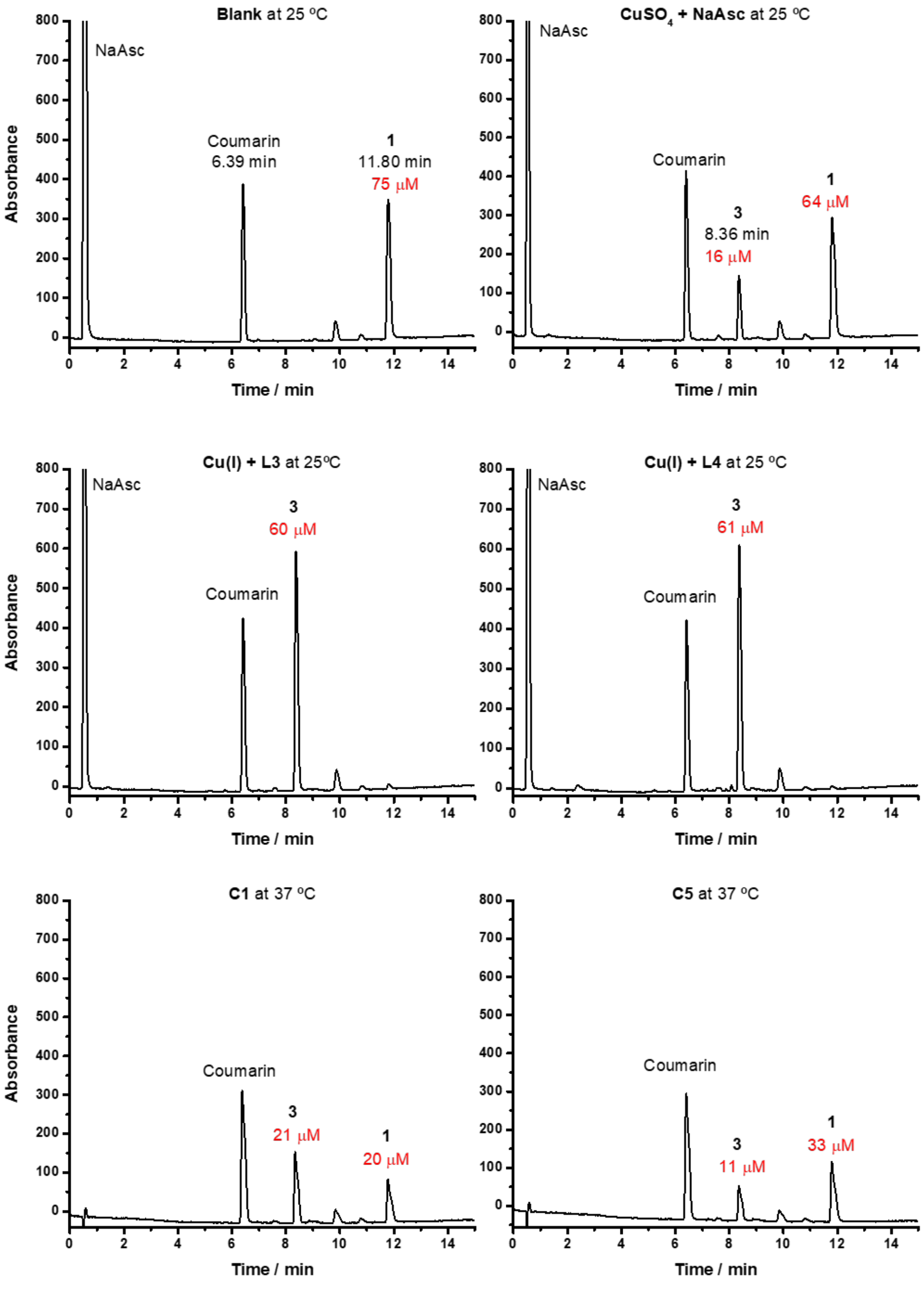

Figure S7. HPLC monitoring of the CuAAC reaction between anthracenyl azide **1** and propargyl alcohol **2** at the indicated temperature

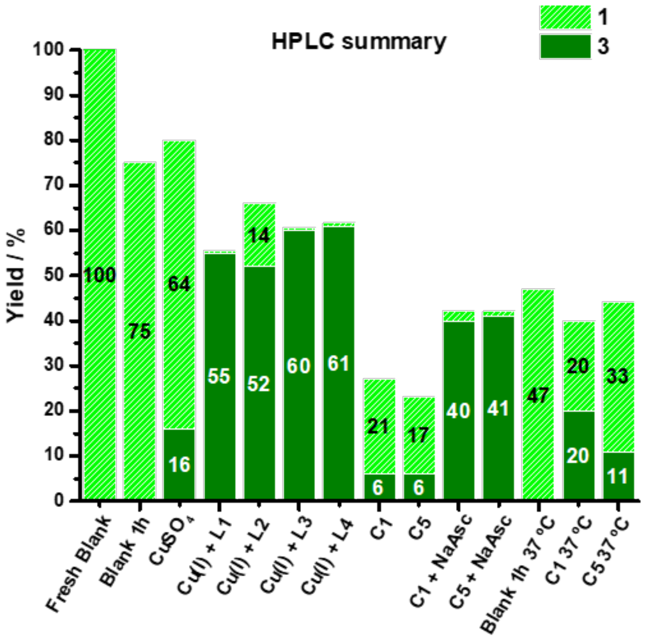

Figure S8. Summary of the CuAAC results obtained using HPLC measurements. Pale green bar corresponds to remaining azide.

S6. Spectroscopic analysis of the copper complexes

6.1 Monitoring the *in situ* formation of Cu(I) complexes with L3

UV-VIS: The complexes were prepared *in situ*, under air, by the sequential mixture of a fresh solution of $CuSO_4$ (0.4 mL, 100 mM in H_2O), **L3** (16.7 mg) and a fresh solution of sodium ascorbate (0.32 mL, 500 mM in H_2O) in a 2 mL eppendorf tube containing 1.28 mL of H_2O. The mixture was stirred for 10 min, for a final concentration of 20 mM $CuSO_4$ (1 equiv), 20 mM **L3** (2 equiv) and 80 mM sodium ascorbate (4 equiv).

For the experiments with 2 mM of copper, the mixture was diluted ten times.

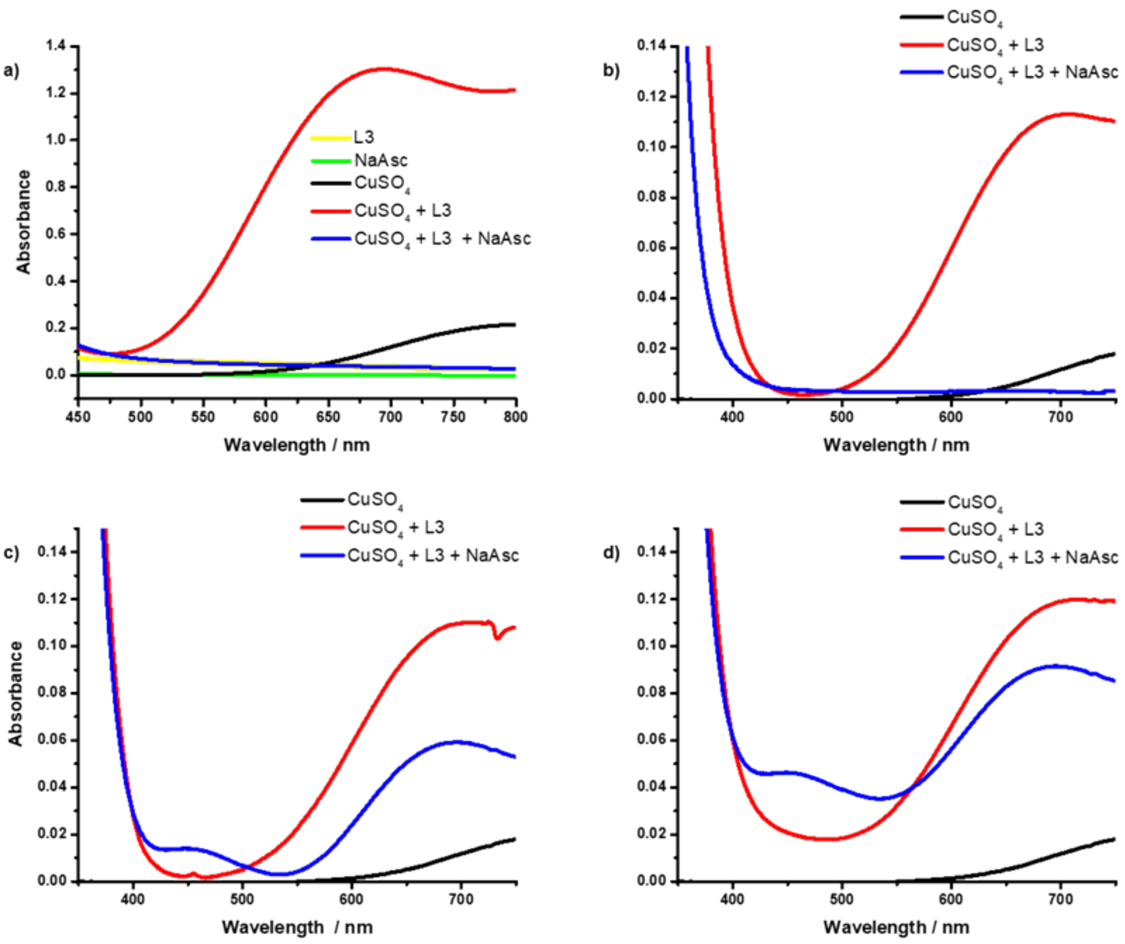

Figure S9. UV spectra at different times. (a) After 10 min of mixing, final concentration of 20 mM of the copper source and **L3**, and 80 mM of NaAsc; (b) After 10 min of mixing, final concentration of the copper source of 2 mM; (c) The same of (b) after 24 h; (d) The same of (b) after seven days.

NMR: The formation of the copper complexes with **L3** was also monitored by NMR. The experiment was performed in individual NMR tubes using D_2O as deuterated solvent at 25 °C:

The complexes were prepared *in situ*, under the air, by the sequential addition in a screw capped vial containing 2 mL of deuterated water, $CuSO_4$ (3.2 mg), **L3** (8.3 mg) and sodium ascorbate (15.8 mg). The final concentration of the mixture is: 10 mM $CuSO_4$ (1 equiv), 10 mM **L3** (1 equiv) and 40 mM sodium ascorbate (4 equiv).

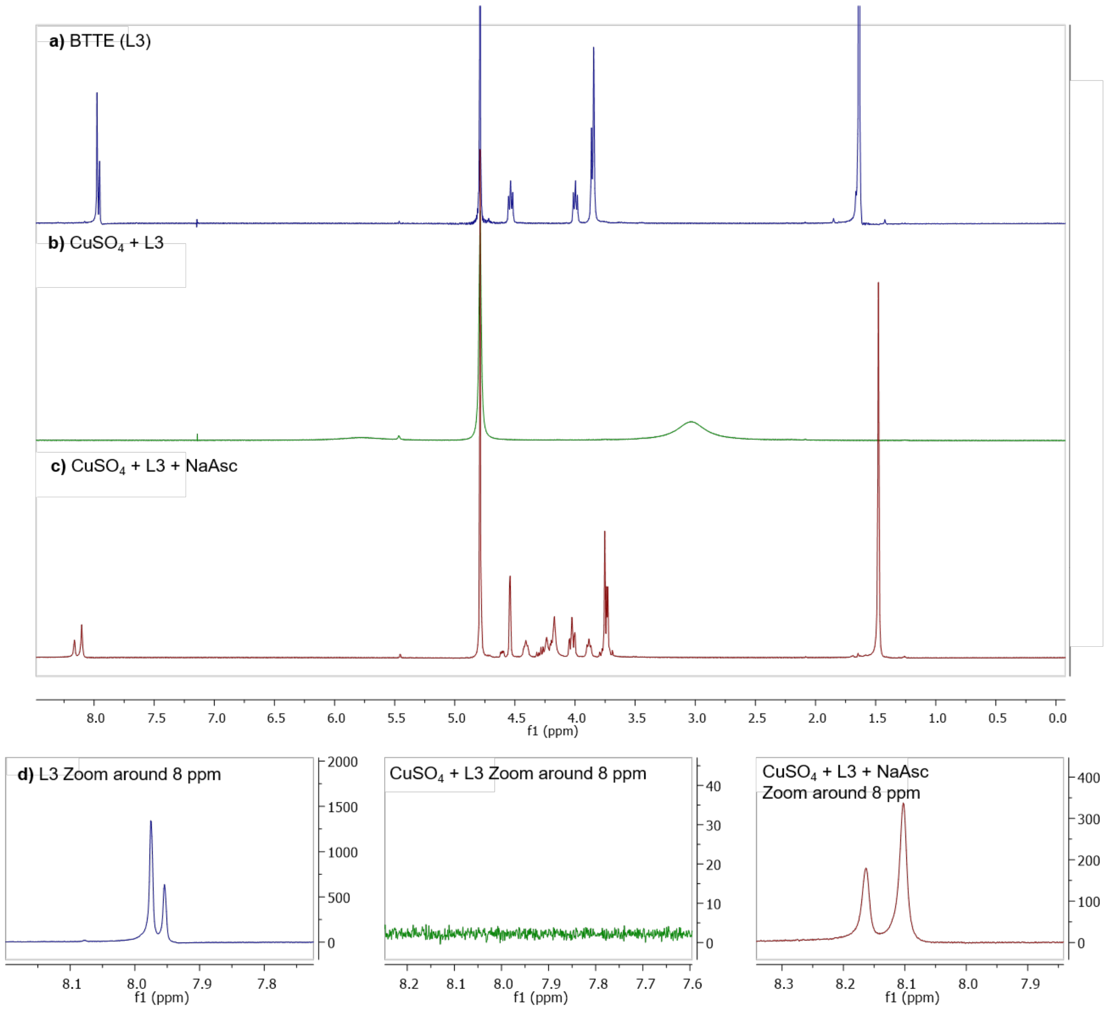

Figure S10. ^{1}H NMR spectra (300 MHz) for the in situ made copper complex using **L3** as ligand in D_2O. (a) Spectrum of **L3** ligand (10 mM, blue line); (b) Spectrum of Cu(II) complex with **L3** (10 mM **L3** and 10 mM of $CuSO_4$, green line); (c) Spectrum of the previous mixture after addition of the reductant (10 mM **L3**, 10 mM of $CuSO_4$ and 40mM of sodium ascorbate, red line). NaAsc was directly added to the D_2O solution containing the ligand **L3** and $CuSO_4$, in the NMR tube under N_2; (d) The triazole proton signal of **L3** ligand reappeared at 8.2-8.1 ppm after addition of NaAsc, confirming the presence of diamagnetic Cu(I) species.

EPR: The studies were performed at 25 °C. For the initial experiment, a solution of $CuSO_4$ (10 mM, H_2O, 1 equiv) was mixed with one equivalent of ligand **L3** (10 mM, H_2O, 1 equiv) and stirred for 10 min. After the EPR measurement at 120 K, 1.1 equiv of sodium ascorbate (25 mM in H_2O) was added, and the EPR immediately measured. Another measurement was made after addition of other 0.9 equiv of sodium ascorbate.

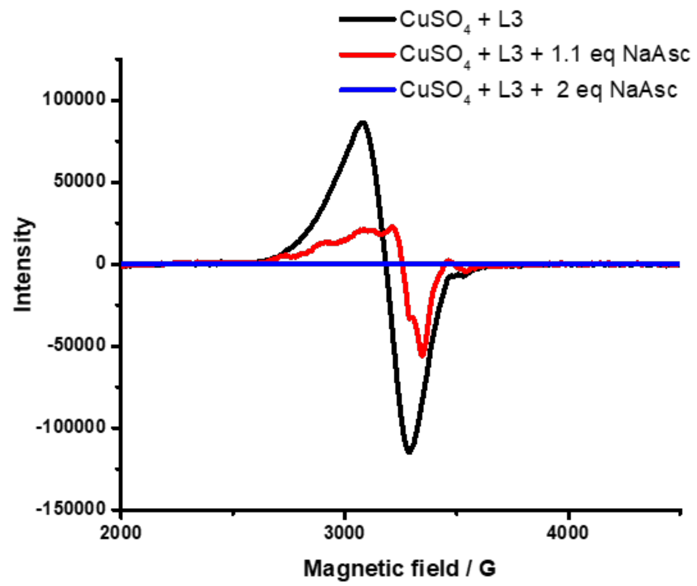

Figure S11. EPR studies of the formation of copper complexes with **L3**.

6.2 Cu(I) complexes C1 and C5. Comparison of their stability by EPR measurements

An initial solution of 10 mM of complexes **C1** or **C5** complexes in DMSO was used for the measurements, without paying any special effort to prevent the presence of air. Samples were taken from this solution at increasing times and frozen in an EPR capillary with liquid nitrogen.

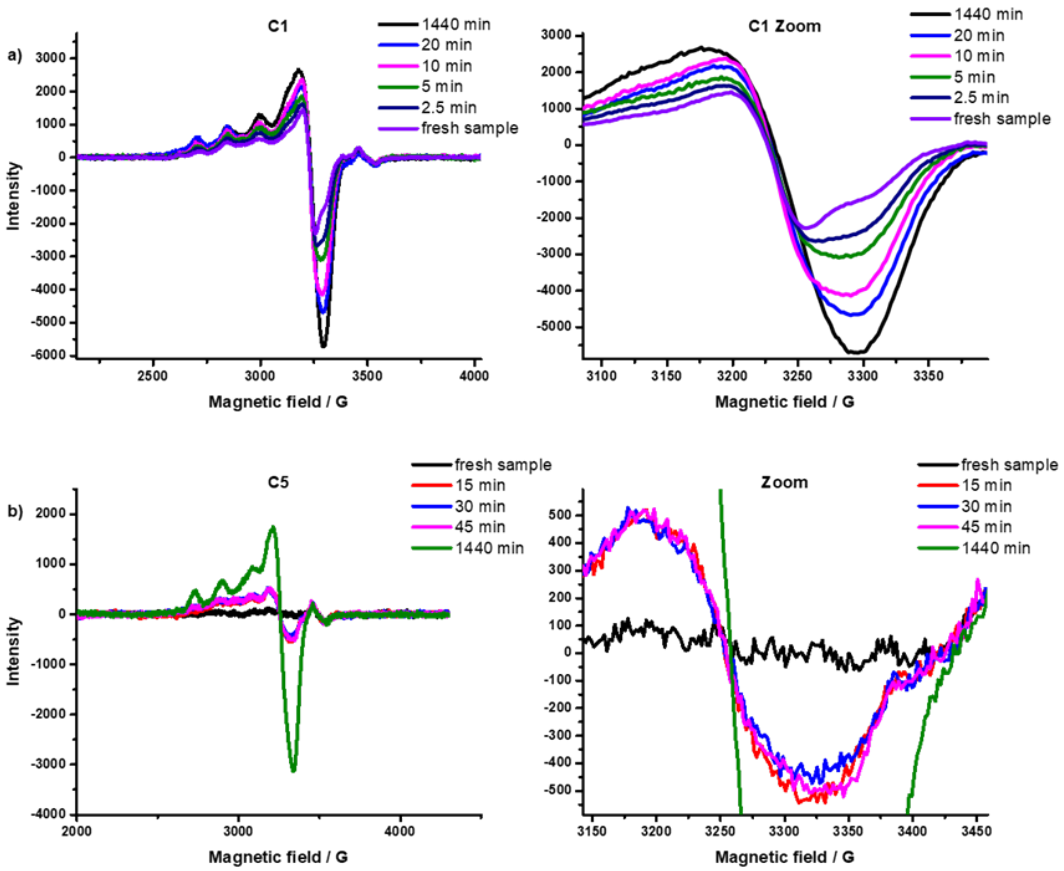

Figure S12. (a) EPR measurements of **C1** and enlarged image; (b) EPR measurements of **C5** and enlarged image.

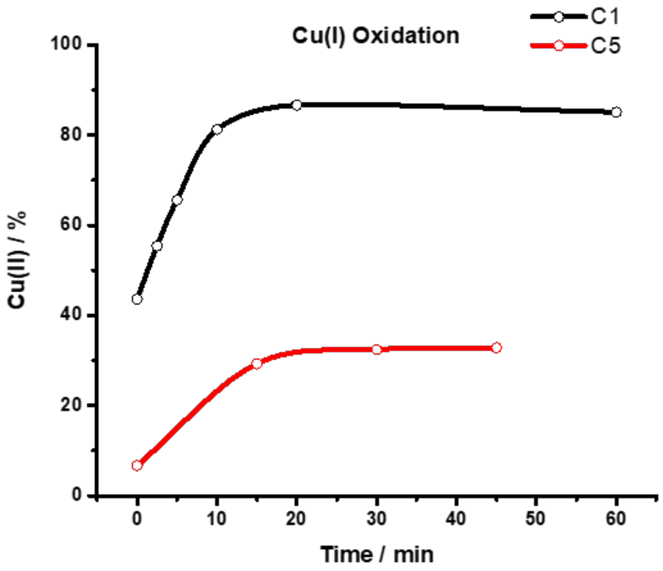

Figure S13. Time dependence of the oxidation of the copper (I) complexes **C1** and **C5**. The sample after 24 h was taken as reference for a complete oxidation of the copper complex (100 % of Cu(II) content), which was further corroborated by NMR.

NMR experiments carried out at time zero, and after 24 h, confirmed the full conversion to the oxidized species after that time, with the disappearance of the signals owing to the paramagnetic behavior of Cu(II). Samples of 24 h were prepared without any taking any special precaution to avoid the oxidation by air.

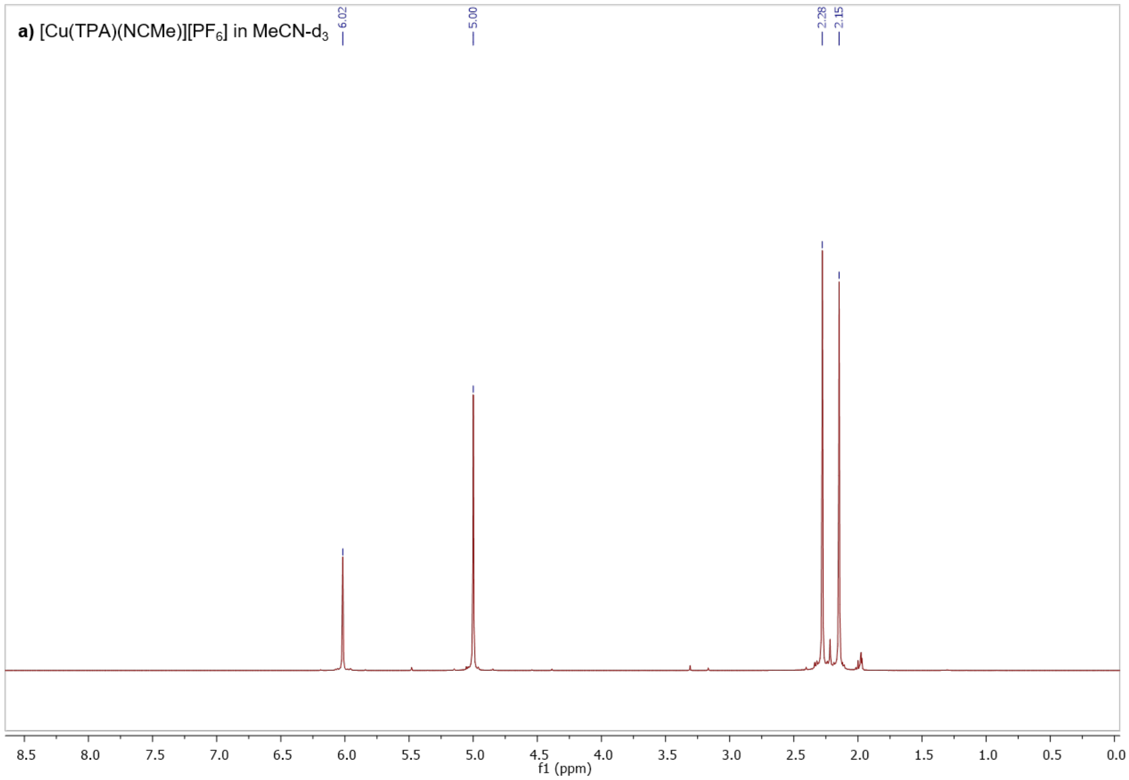

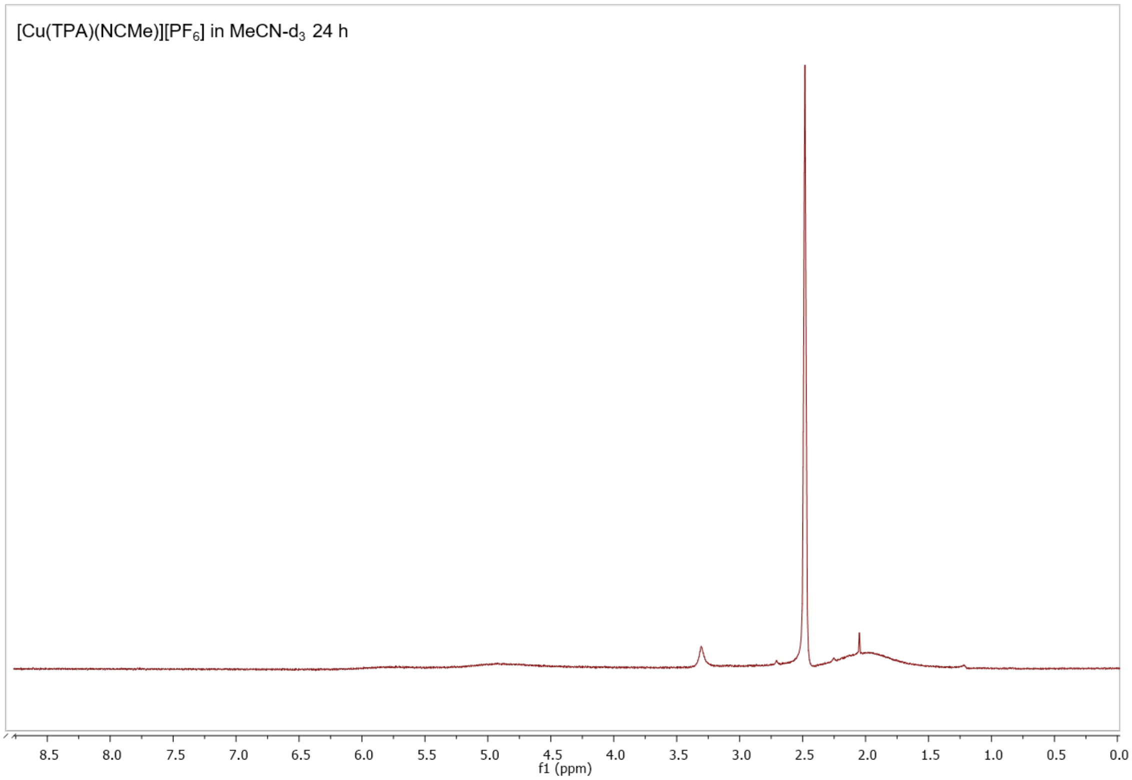

[Cu(TPA)(NCMe)][PF6] in MeCN-d3 24 h
8.5 8.0 7.5 7.0 6.5 6.0 5.5 5.0 4.5 4.0 3.5 3.0 2.5 2.0 1.5 1.0 0.5 0.0
f1 (ppm)

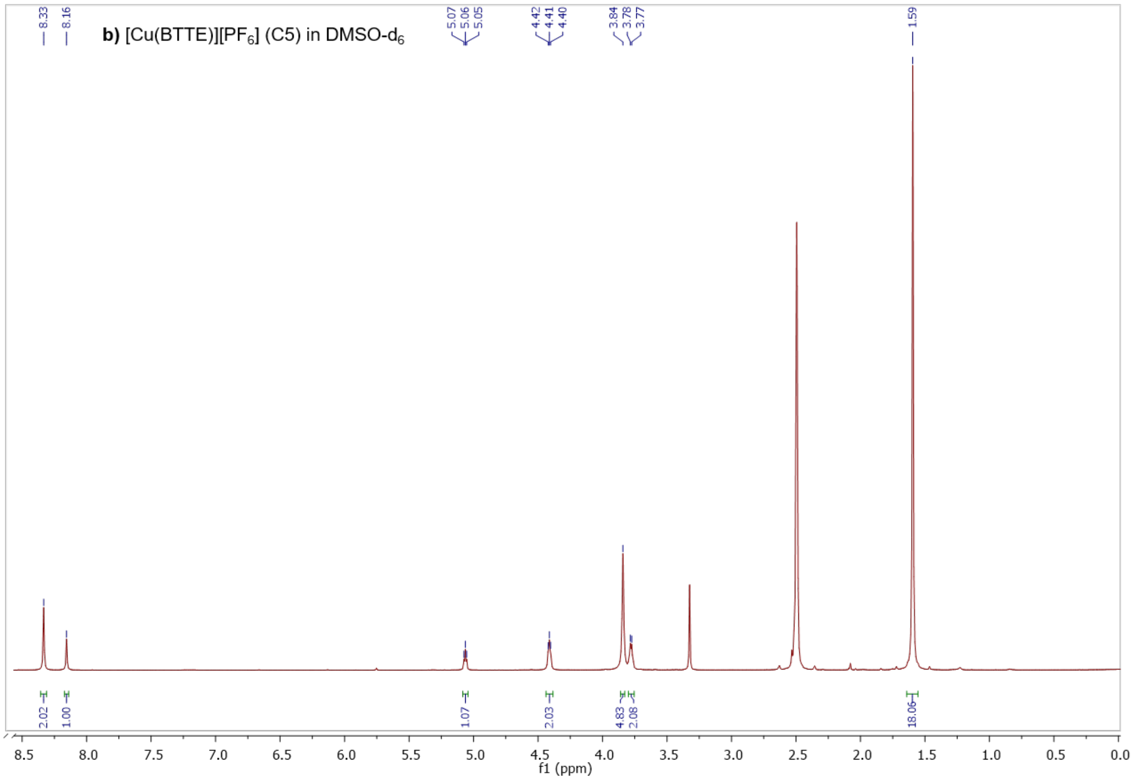

8.33
8.16
b) [Cu(BTTE)][PF6] (C5) in DMSO-d6
5.07
5.06
5.05
4.42
4.41
4.40
3.84
3.78
3.77
1.59
2.02
1.00
1.07
2.03
4.83
2.08
18.06
8.5 8.0 7.5 7.0 6.5 6.0 5.5 5.0 4.5 4.0 3.5 3.0 2.5 2.0 1.5 1.0 0.5 0.0
f1 (ppm)

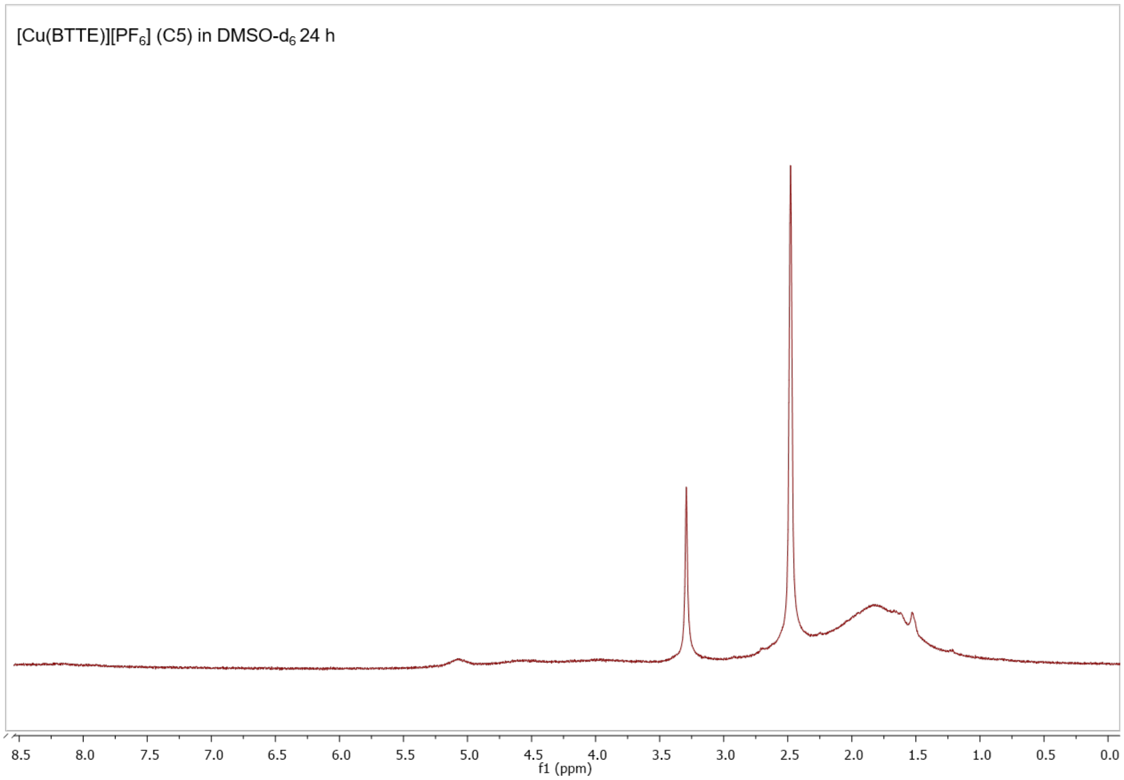

Figure S14. NMR spectra of solutions of complex **C1** (a) and complex **C5** (b) at the beginning and after 24 h.

S7. NMR spectra

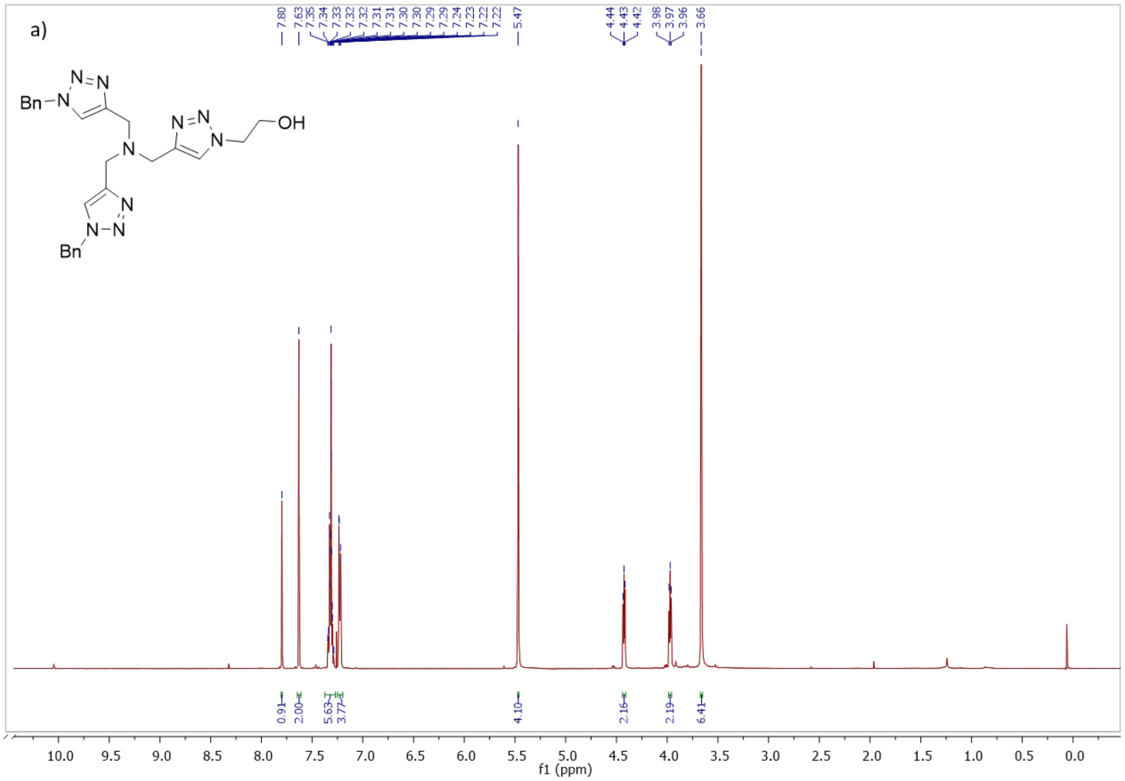

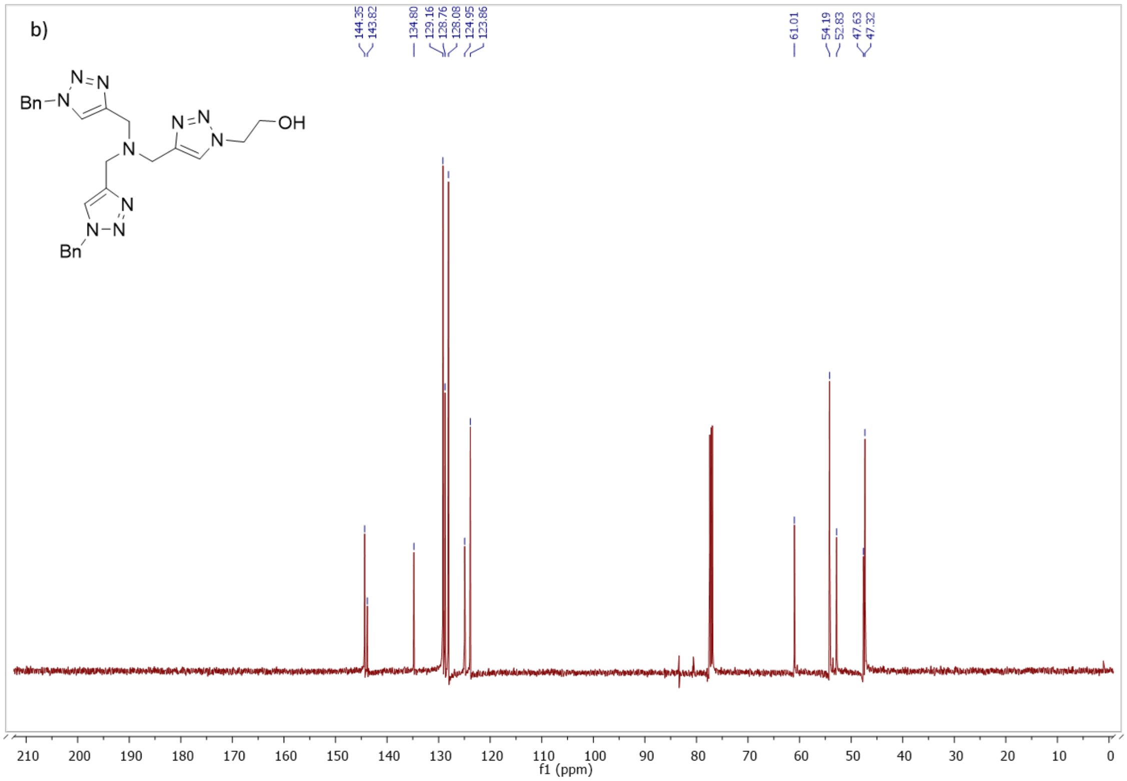

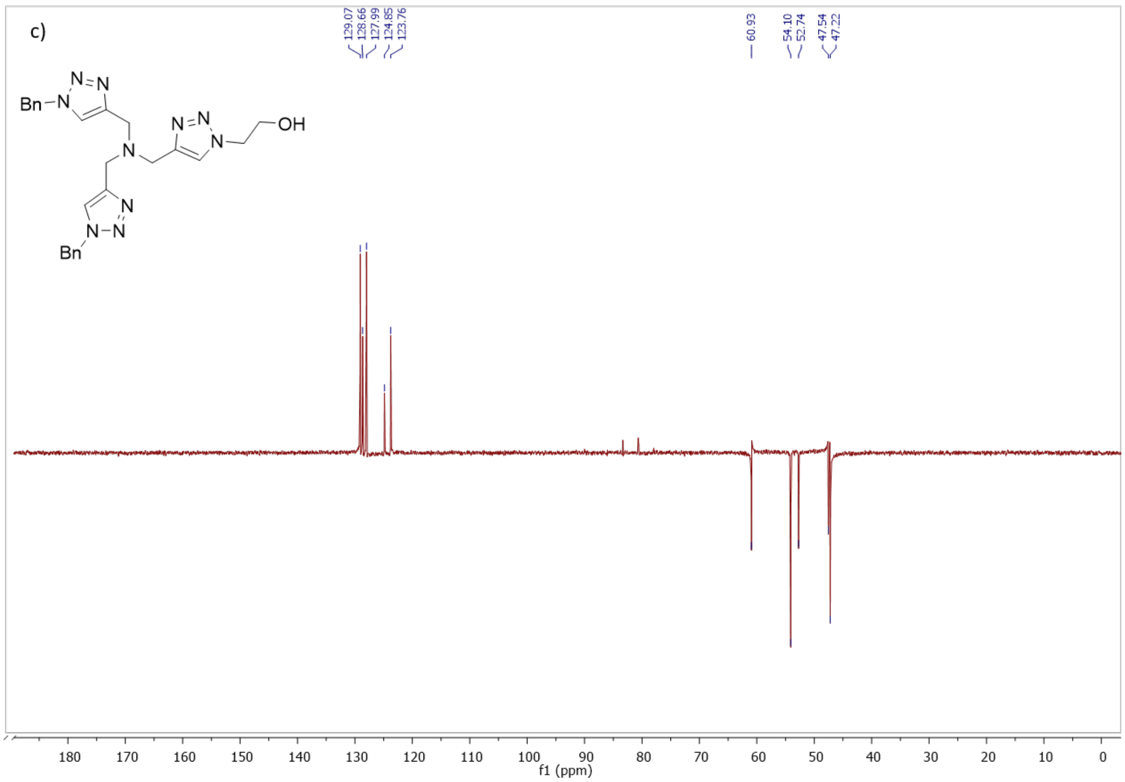

Figure S15. (a) ^{1}H NMR; (b) ^{13}C NMR; (c) DEPT NMR of **L2**.

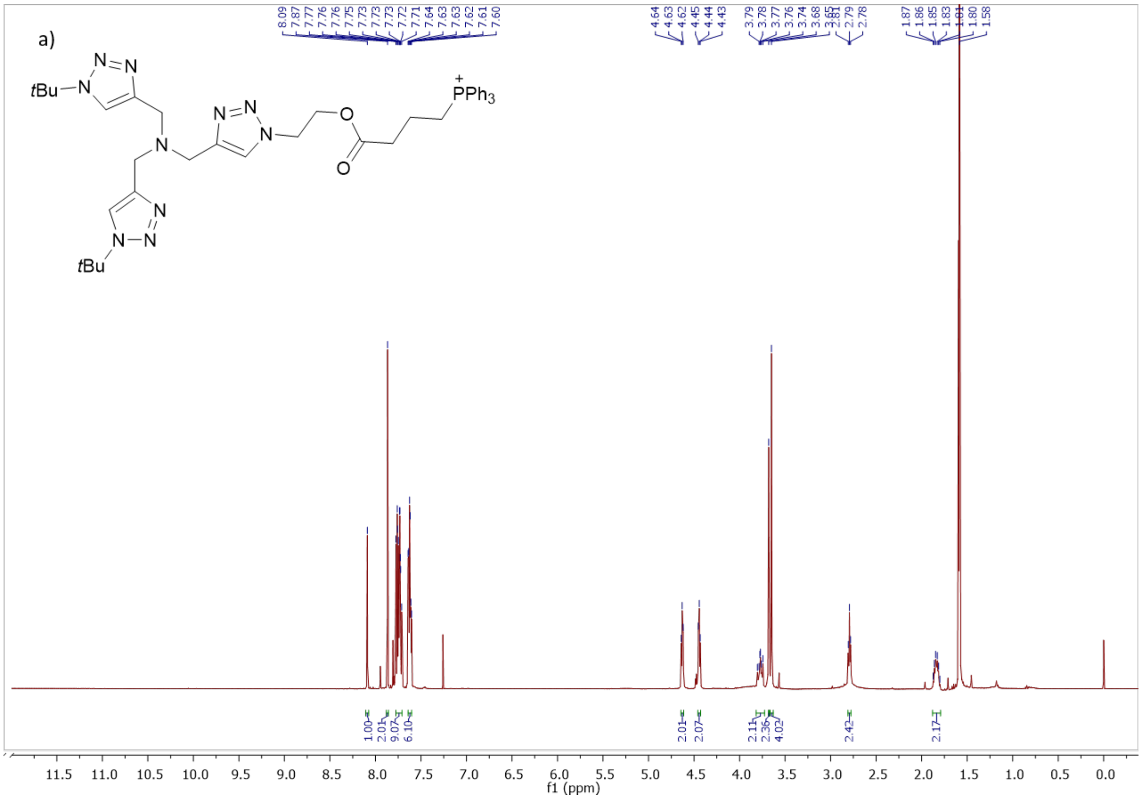
a)
8.09 7.87 7.77 7.76 7.76 7.75 7.73 7.73 7.73 7.72 7.71 7.64 7.63 7.63 7.62 7.61 7.60
4.64 4.63 4.62 4.45 4.44 4.43
3.79 3.78 3.77 3.76 3.74 3.68 3.65 2.81 2.79 2.78
1.87 1.86 1.85 1.83 1.81 1.80 1.58
1.00 2.01 9.07 6.10 2.01 2.07 2.11 2.36 4.02 2.42 2.17
f1 (ppm)

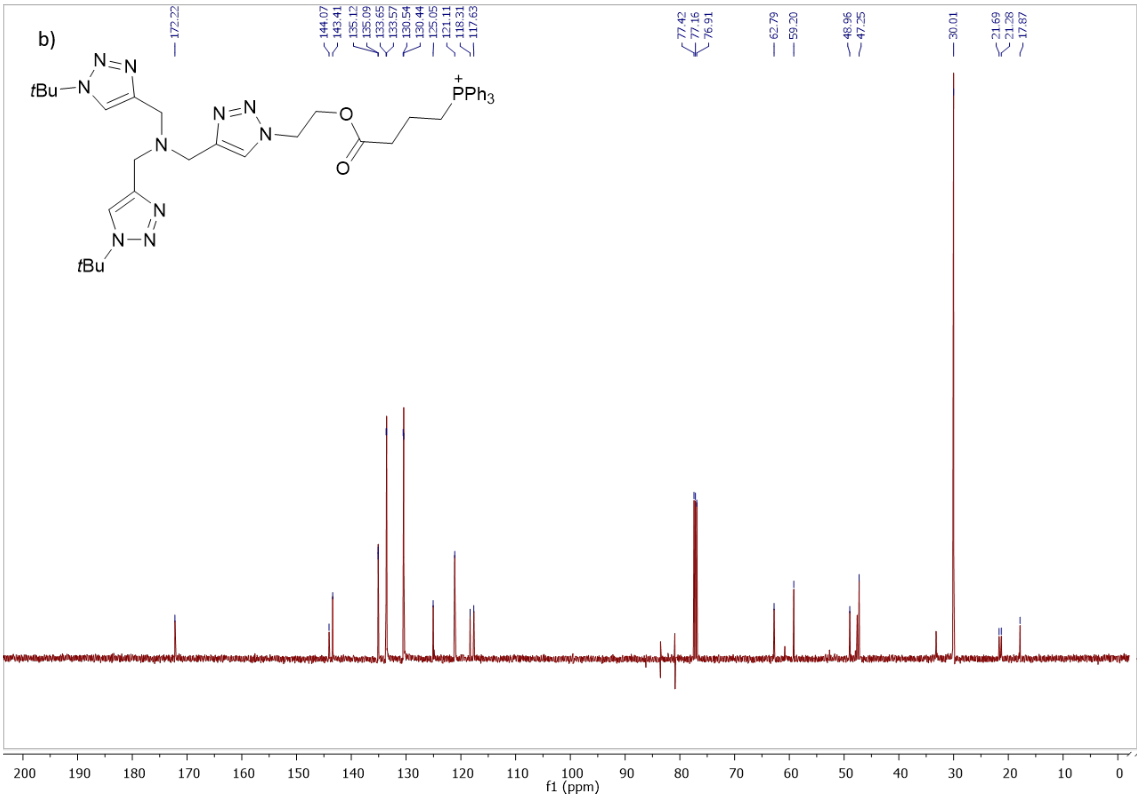
b)
172.22
144.07 143.41 135.12 135.09 133.65 133.57 130.54 130.44 125.05 121.11 118.31 117.63
77.42 77.16 76.91
62.79 59.20
48.96 47.25
30.01
21.69 21.28 17.87
f1 (ppm)

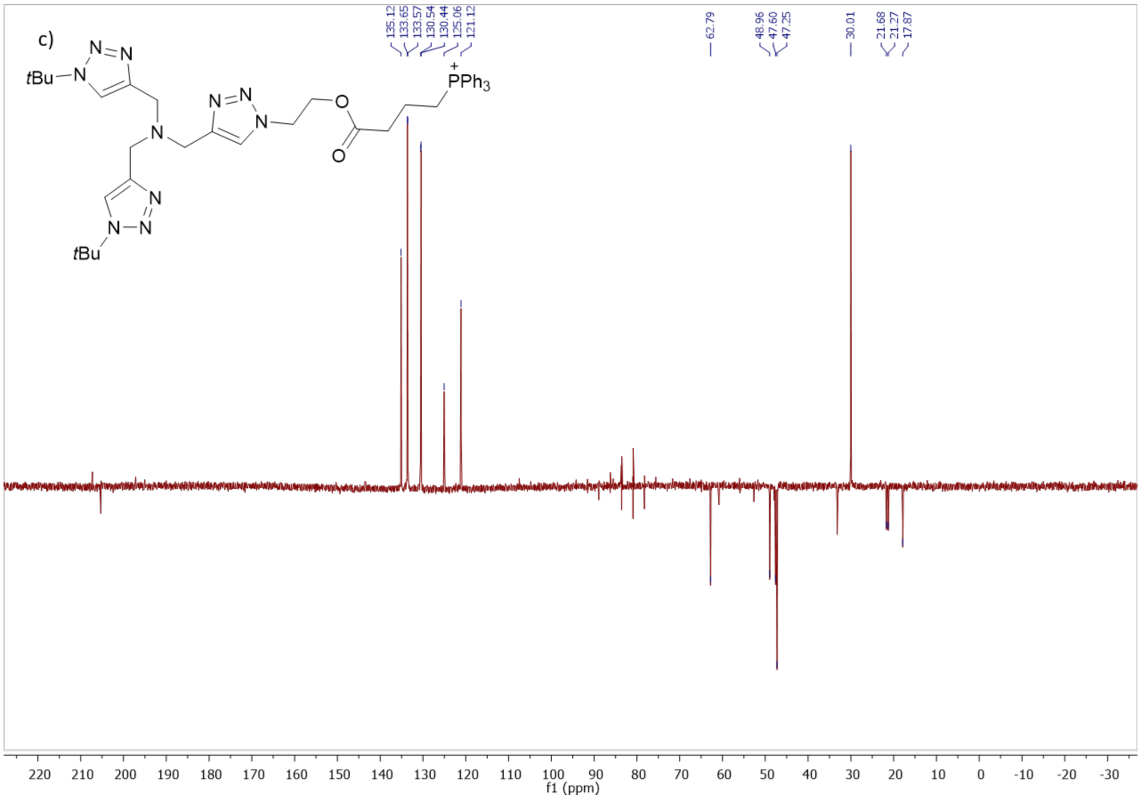

Figure S16. (a) ^{1}H NMR; (b) ^{13}C NMR; (c) DEPT NMR of **L4**.

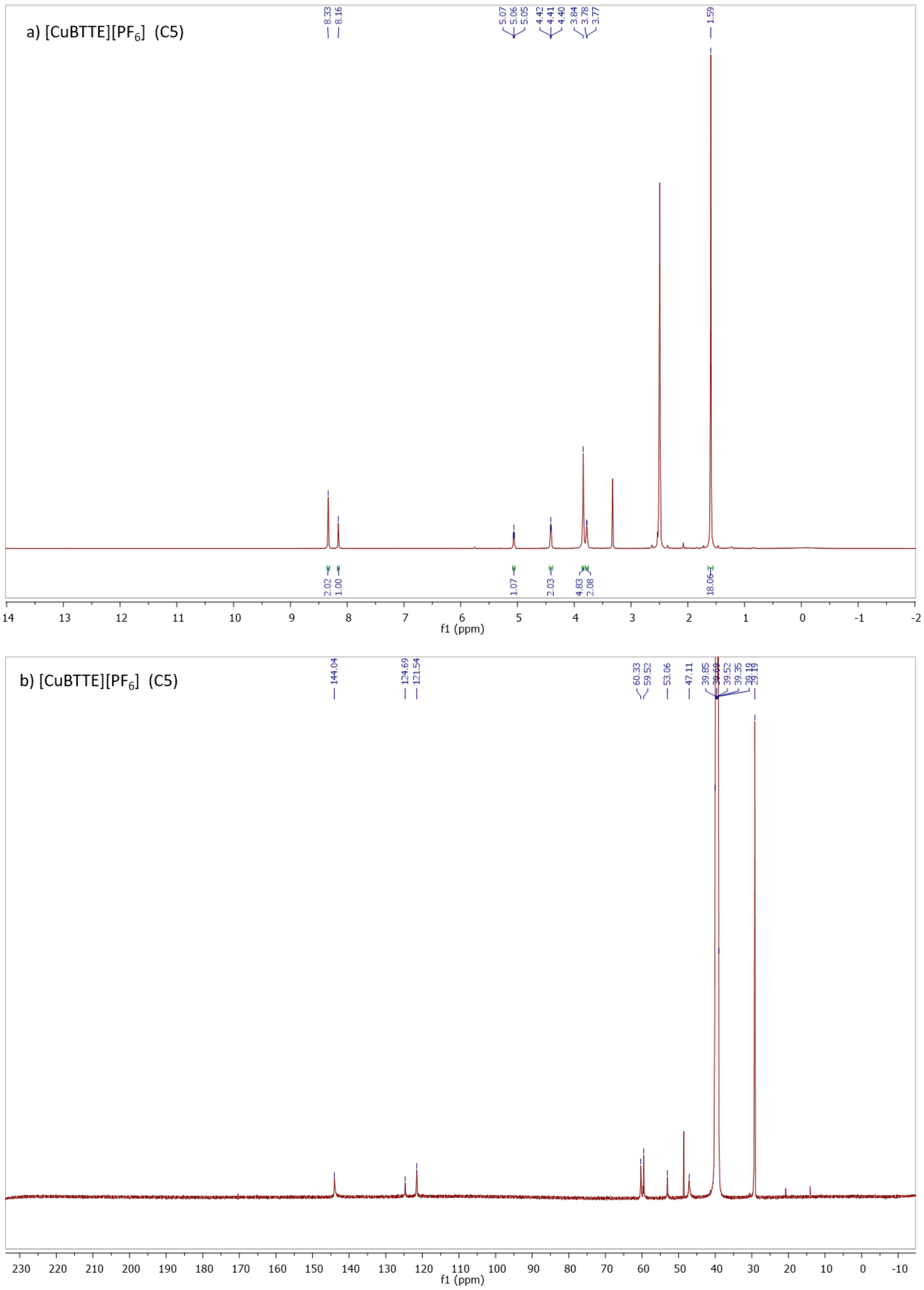

Figure S17. (a) ^{1}H NMR; (b) ^{13}C NMR of **C5**.

S8. General information for the biological experiments

All steps were performed on a sterile clean bench *Tesltar AV-100* at room temperature. Solutions stored in a fridge were warmed beforehand in a water bath (37 °C). Unless otherwise specified, all incubations were performed in DMEM.

Cell Culture: All cell lines were cultured in DMEM (Dulbecco´s modified Eagle´s medium), 5 mM glutamine, penicillin (100 units mL^{-1}) and streptomycin (100 units mL^{-1}) (all from Invitrogen). Proliferating cultures were maintained in a 5% CO_2 humidified incubator at 37 °C.

Fluorescence microscopy: All images were obtained with an *Andor Zyla* mounted on a *Nikon TiE*. Images were further processed with Image J or NIS software (Nikon).

Microscopy settings: The parameters of the fluorescent channels were the following:

For the Nikon (Semrock): filter cube DAPI-1160B-000: BP 387/11 nm, LP 447/60 nm and DM 409 nm; filter cube FITC-3540C-000: BP 482/35 nm, LP 536/40 nm and DM 506 nm; filter cube TRITC-B-000: BP 543/22 nm, LP 593/40 nm and DM 562 nm.

Cytometry studies: The studies were performed in a BD Accuri C6 Flow Cytometer. Data were further processed with FlowJo_V10.

S9. CuAAC experiments in living cells

9.1. Reactions with *in situ* prepared complexes containing ligands L1-L4

The complexes were prepared as follows: in a 500 µL eppendorf tube were successively added 52 µL of MilliQ-H_2O, 1.875 µL of $CuSO_4$ (100 mM in H_2O) and 18.75 µL of the corresponding ligand (20 mM in H_2O). The solution was kept 1 h at room temperature. Then, 2.34 µL of sodium ascorbate (500 mM in H_2O) were added to the solution, and the mixture incubated for 30 min at room temperature. The final concentration of copper was 2.5 mM.

Cells growing on glass coverslips were incubated with the *in situ* made copper complex with ligands **L1-L4** (12 µL of the above solution) in 388 µL of DMEM for 30 minutes, for a final copper concentration of 75 µM. Cells were then washed twice with 400 µL of DMEM and incubated with a solution of propargyl alcohol (**2**) (200 µM) and anthracenyl azide (**1**) (100 µM) in 400 µL of DMEM for 1 hour. Prior to observation by fluorescence microscopy, the samples were washed twice with 400 µL of DMEM. The coverslips were observed “in vivo” in a fluorescence microscope equipped with adequate filters. Digital pictures of the different samples were taken under identical conditions of gain and exposure. The micrographies displayed in Figure S19 corroborate the results reported in the main text.

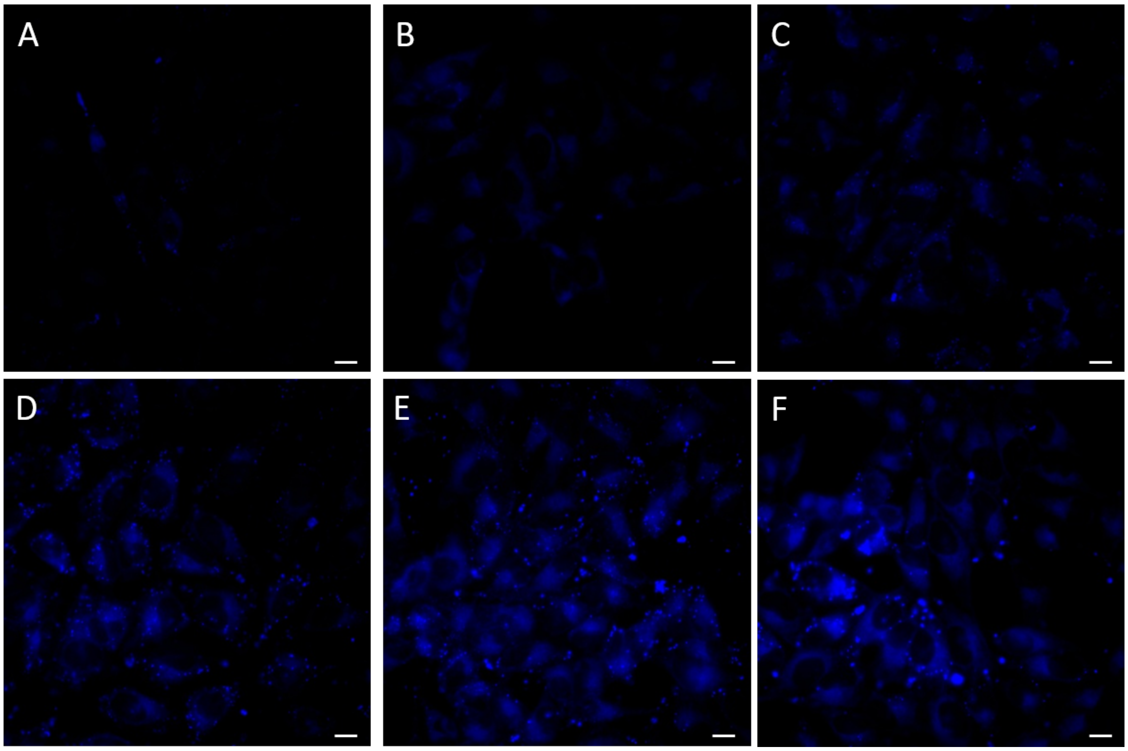

Figure S18. Fluorescence micrographies in experiments carried out using in situ preformed Cu(I)/**Ln** species and HeLa cells. The *in situ* made copper(I) complexes were made by mixing 75 μM $CuSO_4$, 2 equiv Ln, and 6 equiv NaAsc for 10 min. Reaction conditions: Cells were incubated with the copper containing mixture (75 μM) for 30 minutes, followed by two washings with DMEM (2 x 5 min) and treatment with substrates **1** (100 μM) and **2** (200 μM) for 1 h, followed by double washing with DMEM (2 x 5 min). A: Control experiment with cells incubated with azide **1** (100 μM) and alkyne **2** (200 μM) for 1 h, followed by double washing with DMEM (2 x 5 min), without the copper (I) mixture. B-F: Cells after incubation with the copper containing mixtures (30 min), DMEM washings (2 x 5 min), and treatment with **1** (100 μM) and **2** (200 μM) for 1 h, followed by double washing with DMEM (2 x 5 min). B: without ligands. C: with **L2**. D: with **L3**. E: with **L1**. F: with **L4**.. Basal levels of fluorescence were normalized by LUT equalization. Scale bar, 12.5 mm.

9.2. Reactions with the defined complexes C1-C5

A fresh solution of each catalyst (10 mM in DMSO) in a 2 mL eppendorf tube was prepared before the experiment.

Cells growing on glass coverslips were incubated with either catalyst **C1-C5** (50 μM, 2 μL of the fresh solution) in 398 μL of DMEM for 30 minutes. Cells were then washed twice with 400 μL of DMEM and incubated with the mixture of propargyl alcohol (**2**) (200 μM) and anthracenyl azide (**1**) (100 μM) in 400 μL of DMEM for 1 hour. Prior to observation by fluorescence microscopy, the samples were washed twice with 400 μL of DMEM. The coverslips were observed in a fluorescence microscope equipped with adequate filters. Digital pictures of the different samples were taken under identical conditions of gain and exposure. The micrographies displayed in Figure S20 corroborate the results reported in the main text.

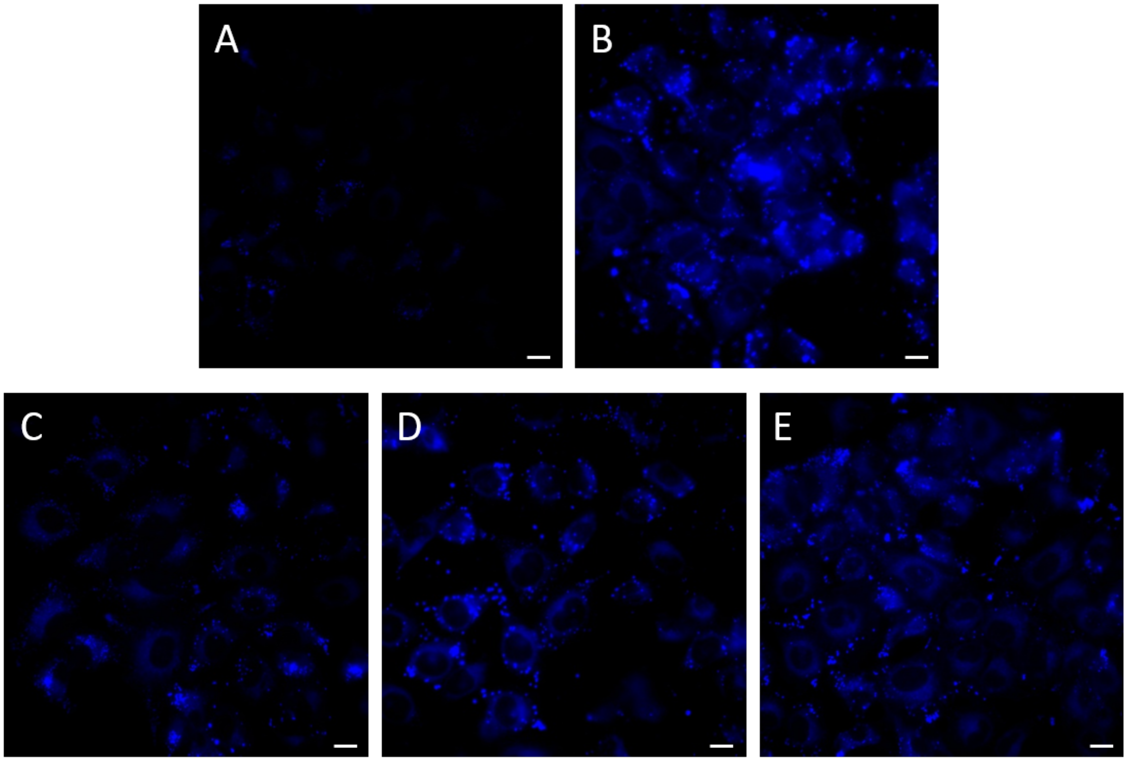

Figure S19. Fluorescence micrographies in experiments carried out using preformed Cu(I) complexes **C1**, **C2**, **C4** and **C5** in A549 cells. A: Control experiment without copper complex; cells incubated with azide **1** (100 µM) and alkyne **2** (200 µM) for 1 h, followed by double washing with DMEM (2 x 5 min). B-E: Cells after incubation with the preformed Cu(I) complex (50 µM) for 30 min, DMEM washings (2 x 5 min), and treatment with **1** (100 µM) and **2** (200 µM) for 1 h, followed by double washing with DMEM (2 x 5 min) (B: **C5**. C: **C1**. D: **C2**. E: **C4**). A 10 mM solution of the Cu(I) complexes in DMSO was freshly prepared and used immediately to avoid oxidation of the complex. Basal levels of fluorescence were normalized by LUT equalization. Scale bar, 12.5 mm.

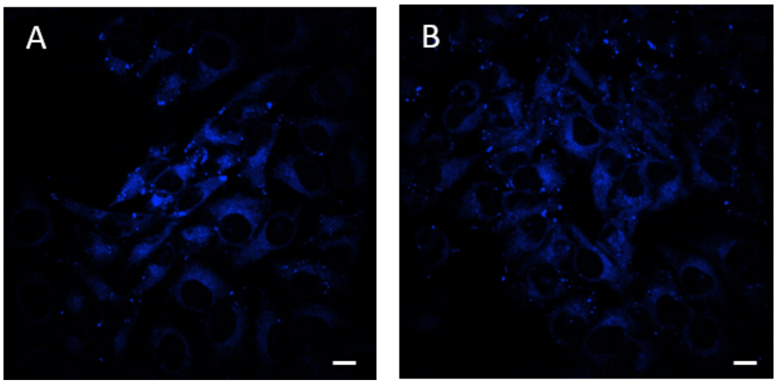

Figure S20. Fluorescence micrographies in experiments carried out using in situ preformed Cu(I)/**L3** and HeLa cells, and the same experiment in presence of EDTA. A: Cells after incubation with the copper containing mixtures (75 µM, 30 min), DMEM washings (2 x 5 min), and treatment with **1** (100 µM) and **2** (200 µM) for 1 h, followed by double washing with DMEM (2 x 5 min). B: Cells after incubation with the copper containing mixtures (75 µM, 30 min), DMEM washings (2 x 5 min), EDTA washing (150 µM, 10 min) and treatment with **1** (100 µM) and **2** (200 µM) for 1 h, followed by double washing with DMEM (2 x 5 min). Basal levels of fluorescence were normalized by LUT equalization. Scale bar, 12.5 mm.

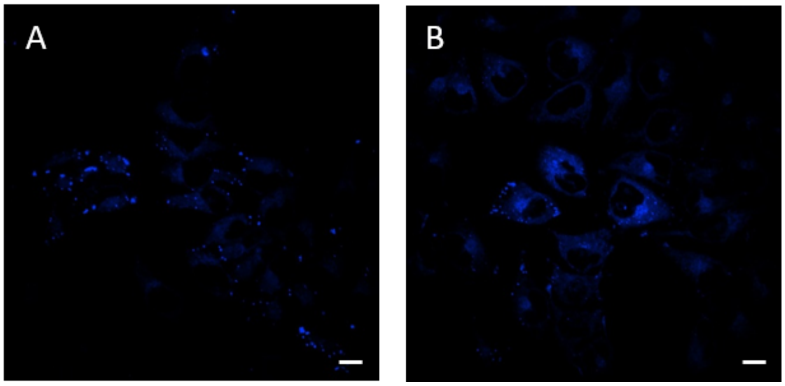

Figure S21. Fluorescence micrographies in experiments carried out using Cu(II)/**L3** species and HeLa cells, without or with a reductant. A: Cells after incubation with a premade mixture of $CuSO_4$ and 2 equiv of **L3**, (75 μM in copper, 30 min), DMEM washings (2 x 5 min), and treatment with **1** (100 μM) and **2** (200 μM) for 1 h, followed by double washing with DMEM (2 x 5 min). B: Cells after incubation with the same copper(II) containing mixture (75 μM, 30 min), DMEM washings (2 x 5 min), and addition of NaAsc (450 μM, 20 min), DMEM washings (2 x 5 min) and treatment with **1** (100 μM) and **2** (200 μM) for 1 h, followed by double washing with DMEM (2 x 5 min). Basal levels of fluorescence were normalized by LUT equalization. Scale bar, 12.5 mm.

S10. Viability Assays

The toxicity of the *in situ* made copper complexes with ligands **L1-L4,** as well as of the defined complexes **C1-C5** was tested by MTT assays in HeLa cell line as follows: 150000 cells per well were seeded in 96 well plates one day before treatment with different concentrations of the catalysts in 100 μL of DMEM. After the adequate time of incubation (2 h or 12 h), Thiazolyl Blue Tetrazolium Bromide (*Sigma*) was added to the cell culture medium to a final concentration of 0.5 mg mL^{-1}. Cells were then incubated for 4 h to allow the formation of formazan precipitates by metabolically active cells. A detergent solution of 10% SDS (sodium dodecyl sulfate) and 0.01 M HCl was then added and the plate was incubated overnight at room temperature to allow the solubilization of the precipitates. The quantity of formazan in each well (directly proportional to the number of viable cells) was measured by recording changes in absorbance at 570 nm in a microtiter plate reading spectrophotometer (*Tecan Infinite 200 PRO).*

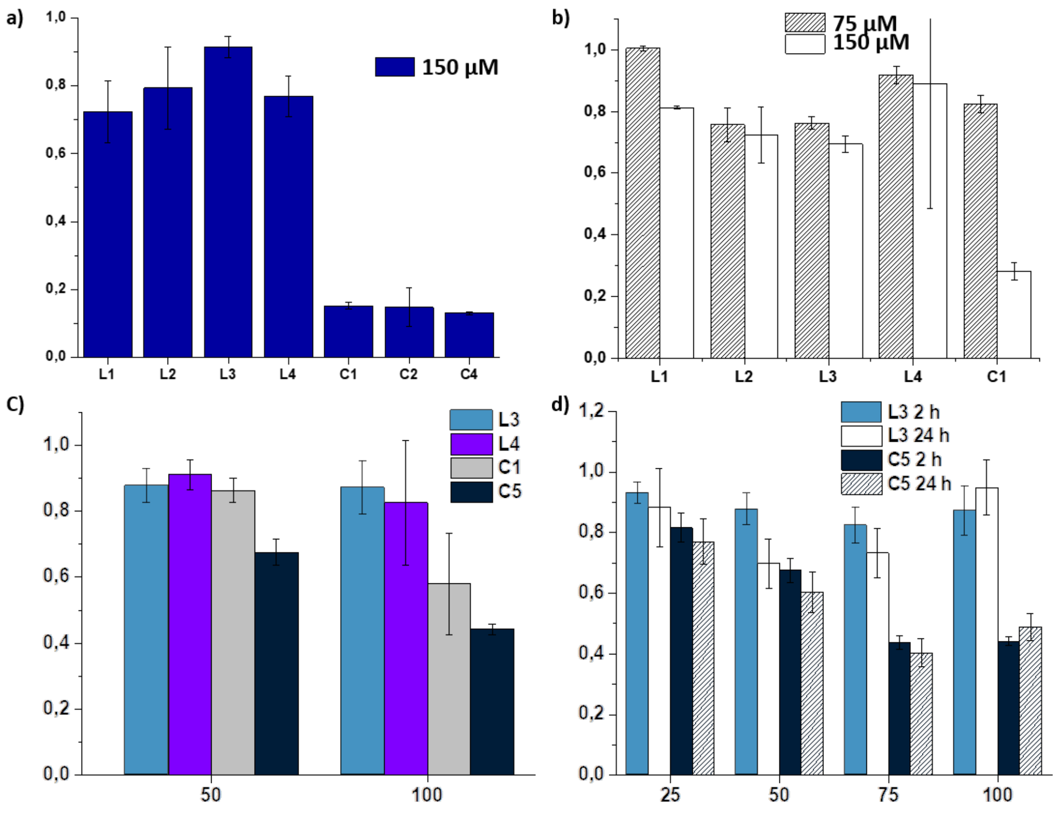

Figure S22. Viability Assays (MTT). HeLa cells were incubated in cell culture medium containing the indicated amounts of the copper complexes: (a) In situ made Cu(I) complexes with **L1**-**L4** and preformed complexes **C1**, **C2** and **C4** for 12 h; (b) In situ made Cu(I) complexes with **L1**-**L4** for 2 h; (c) in situ made Cu(I) complexes with **L3** and **L4** and defined complexes **C1** and **C5,** after 2 h; (d) in situ made Cu(I) complex with **L3** and defined complex **C5** after 2 h and 24 h**.** The viability is expressed as the fold change of the absorbance value with respect to untreated cells (value 1.0).

In assessing these results, it must be taken into account that the amount of copper internalized depends on the complexes, and the toxicity can be in great part associated to such cell entrance.

S11. ICP analysis

For the ICP measurements, a total of 3x10^6 HeLa cells growing in 6 well plates were treated with each copper complex. Each well was incubated with the different *in situ* made copper complexes with **L1-L4** (75 µM, 22.5 µL of the fresh solution) in 728 µL of DMEM, or the different defined Cu(I) complexes **C1-C5** (50 µM, 3.75 µL of the fresh solution) in 747 µL of DMEM for 1 h. Prior to digestion, the samples were washed with 750 µL of DMEM and then twice with 750 µL of PBS. The obtained fractions were digested in duplicate in 500 µL of HNO_3 (70%) by microwave heating and analyzed.

For the membrane protein extraction, after incubation with the copper complexes for 1 h, the samples were washed with 750 µL of DMEM and then twice with 750 µL of PBS. Then a commercial kit (Mem-PER Plus Membrane Protein Extraction Kit -Thermo-Fisher Scientific-) was used to isolate membrane and cytosolic cellular fractions.

Procedure for Membrane Protein Extraction from HeLa cells with Mem-PER Plus Membrane Protein Extraction Kit

3 × 10^6 cells were resuspended in 500 µL of DMEM by scraping the cells off the surface of the plate with a cell scraper. Then, the harvested cell suspension was centrifuged at 300 × *g* for 5 minutes. The cell pellet was washed with 300 µL of Cell Wash Solution and centrifuged at 300 × *g* for 5 minutes.

Carefully the supernatant was removed and discarded. The cells were resuspended in 500 µL of Cell Wash Solution, transferred to a 0.5 mL Eppendorf and centrifuged at 300 × *g* for 5 minutes. The supernatant was discarded. After addition of 150 µL of Permeabilization Buffer to the cell pellet, the eppendorf was vortexed briefly to obtain a homogeneous cell suspension and then incubated 10 minutes at 4 °C with constant mixing. The permeabilized cells were centrifuged for 15 minutes at 16,000 × *g*. The supernatant containing cytosolic proteins was carefully removed and transferred to a new eppendorf. Then, 200 µL of Solubilization Buffer were added to the pellet and resuspended by pipetting up and down. The eppendorfs were incubated at 4 °C for 30 minutes with constant mixing. Finally, the eppendorfs were centrifuged at 16,000 × *g* for 15 minutes at 4 °C and the supernatant containing solubilized membrane and membrane-associated proteins was transferred to a new eppendorf. The obtained fractions were digested in duplicate in HNO_3/H_2O_2 by microwave heating and analyzed.

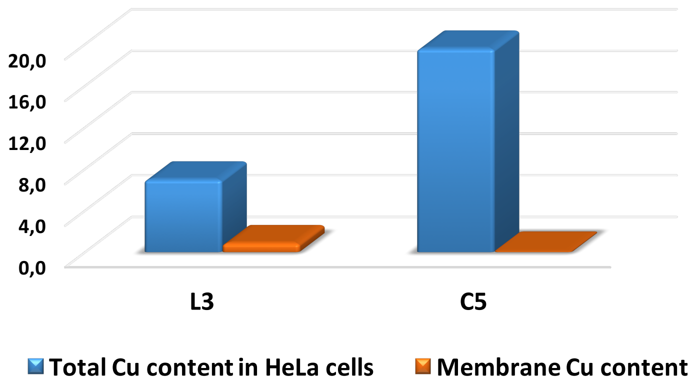

Figure S23. ICP-MS results of the copper accumulated in the membrane of HeLa cells.

S12. Flow cytometry studies of the reactivity

Cytometry studies were performed in HeLa cells: 200000 cells per well were seeded in 96 well plates one day before treatment. Then, it was followed the same protocol described for the experiments in living cells, scaling the experiment to a final volume of 100 µL of DMEM. After the incubation time, cells were washed twice with 100 µL of PBS, harvested with 100 µL of trypsin/EDTA for 15 min and resuspended in 2% FBS in 100 µL of PBS buffer with 5 mM EDTA. The fluorescence was analyzed by flow cytometry. As observed in Figure 5 of the main text, quantification by flow cytometry showed clear presence of the product using the violet laser and the emission filter (450/50).

S13. Quantification of the reaction inside living cells

General Procedure: The CuAAC reaction of **1** and **2** with different copper complexes in living cells was quantified by fluorescence measurements using a microplate reader.

The experiments were performed in plates of 12 wells as follows:

100000 cells per well were seeded in 12 well plated two days before treatment with the copper complex and the substrates. For each complex the experiment was done by triplicate.

To normalize the results, number of cells was measured after the experiments were performed. For this, after the incubation time with each complex, cells were washed twice with 400 µL of PBS, harvested with 100 µL of trypsin/EDTA for 5 min at 37 °C and resuspended in 300 µL of DMEM buffer. An aliquot of 50 uL was transferred to an 0.500 mL Eppendorf and diluted with 450 µL of PBS. Then cells were measured with *Scepter™ 2.0 Handheld Automated Cell Counter*.

1. Reactions with *in situ* prepared complexes containing ligand L3

The complexes were prepared as follows: in a 500 μL eppendorf tube were successively added 52 μL of MilliQ-H_2O, 1.875 μL of $CuSO_4$ (100 mM in H_2O) and 18.75 μL of **L3** (20 mM in H_2O). The solution was kept 1 h at room temperature. Then, 2.34 μL of sodium ascorbate (500 mM in H_2O) were added to the solution, and the mixture incubated for 30 min at room temperature. The final concentration of copper was 2.5 mM.

Cells growing on plates of 12 wells were incubated with the *in situ* made copper complex with ligand **L3** (8 μL of the above solution) in 392 μL of DMEM for 30 minutes, for a final copper concentration of 75 μM. Cells were then washed twice with 400 μL of DMEM and incubated with a solution of propargyl alcohol (**2**) (200 μM) and anthracenyl azide (**1**) (100 μM) in 400 μL of DMEM for 1 hour. Prior to lisation, the samples were washed with 400 μL of DMEM followed by two washes with PBS (400 μL). Then 100 μL of a solution of 80% of MeOH in water were added to each well. After 5 min and pipetting up they were transferred to a microplate of 96 well.

Cells were also incubated only with substrates **1** and **2** for 1 h. These cells were treated in the same way and the lysates were used as negative control, to eliminate the fluorescent background signal.

The results were analyzed by fluorescence measurements in a microplate reader (*Tecan infinite F200Pro*). Fluorescence data were obtained by using an excitation wavelength of 360 nm and recorded at 465 nm. Every measured value is the mean value of three aliquots.

2. Reactions with the defined complex C5

A fresh solution of each catalyst (10 mM in DMSO) in a 2 mL eppendorf tube was prepared before the experiment.

Cells growing on plate of 12 wells were incubated with catalyst **C5** (25-50 μM, 2 μL of the fresh solution) in 398 μL of DMEM for 30 minutes. Cells were then washed twice with 400 μL of DMEM and incubated with the mixture of propargyl alcohol (**2**) (200 μM) and anthracenyl azide (**1**) (100 μM) in 400 μL of DMEM for 1 hour. Prior to lisation, the samples were washed with 400 μL of DMEM followed by two washes with PBS (400 μL). Then 200 μL of a solution of 80% of MeOH in water were added to each well. After 5 min and pipetting up they were transferred to a microplate of 96 well.

3. Calibration

Calibration experiments were performed in a 96 wells plate by addition of increasing quantities of a fresh solution of 1-(9-anthracenylmethyl)-1*H*-1,2,3-triazole-4-methanol **3** (5mM in DMSO) in a solution of 80% of MeOH in water. Higher concentration results in the precipitation of the triazole **3**. The experiments were performed by triplicate, and measured in the fluorescence microplate reader (excitation: 360 nm, emission: 465 nm). The fluorescence results were processed with OriginPro 2016 to obtain the calibration equation and applied to quantify the fluorescence of the experiments in living cells.

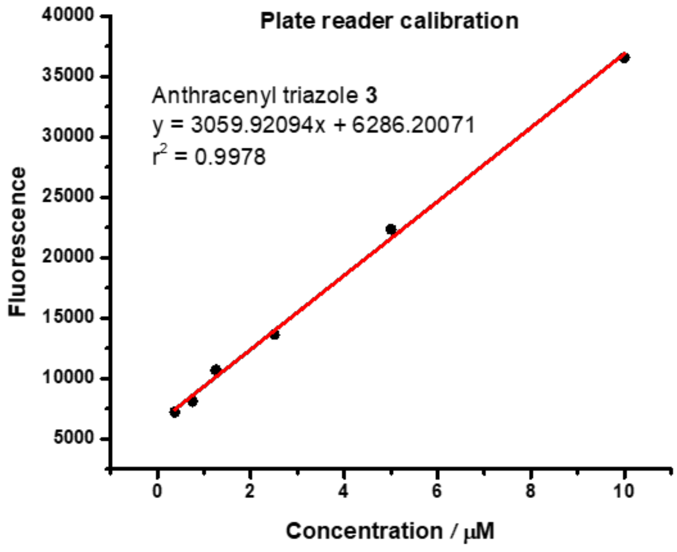

Figure S24. Standard curve of triazole **3**. Fluorescence intensity (λ_{ex} = 360 nm, λ_{em} = 465 nm) at different concentrations. Linear regression curve for 0–10 µM is shown.

4. Cellular uptake of substrate 1

To calculate the amount of substrate **1** that is internalized by the cell the following experiment was performed:

The experiments were performed in plates of 100 mm as follows:

100000 cells per well were seeded in 100 mm plated two days before treatment with substrate **1**. For each measurement, four plates were used.

Cells growing on plate of 100 mm were incubated with the substrate **1** (1.2 µL) in 4 mL of DMEM for 1 hour. Prior to lysis, the samples were washed with 1 mL of DMEM followed by two washes with PBS (1 mL). Then 1 mL of a solution of 80% of MeOH in water was added. After 5min and pipetting up this solution was transferred to a 1.5 mL Eppendorf. Finally, we obtained 4 mL of lysates from the four plates we used. To 982.6 µL of this sample was added 17.4 µL of a stock solution of coumarin 50 mM as internal standard. The sample (10 uL) was injected in the HPLC-UV. Method: H_2O/MeCN gradient 95/5 ⟶ 5/95 in 12 min, then 3 min 100% MeCN.

<u>The calibration</u> of azide **1** was performed in the same HPLC vial, using MeCN as solvent. The different samples were prepared from a 5 mM fresh stock solution of the analyte in DMSO. Coumarine (870 µM as final concentration) was used as internal standard. The areas obtained from the chromatogram were processed with OriginPro 2016 to obtain the calibration equation and applied to quantify the absorbance of the in vitro experiments. No differences were appreciated when MeOH or MeCN were used as solvent.

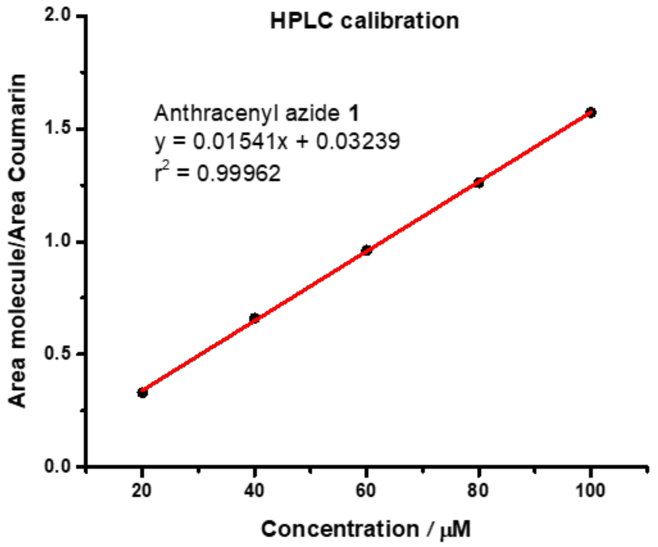

Figure S25. Standard curve of azide **1**. Linear regression curve for 0-100 μM is shown.

To normalize the results, number of cells was measured after the experiments were performed. For this, after the incubation time, cells were washed twice with 1 mL of PBS, harvested with 1 mL of trypsin/EDTA for 5 min at 37 °C and resuspended in 3 mL of DMEM buffer. An aliquot of 50 uL was transferred to an 0.500 mL Eppendorf and diluted with 450 μL of PBS. Then cells were measured with *Scepter™ 2.0 Handheld Automated Cell Counter*.

EXAMPLE:

Number of cells per well in experiments with copper complexes: 428300 cells/mL in 0.4 mL (171320 cells per well)

Uptake of substrate by 2522000 cells/mL in 4 mL: 15 % (59 μM out of 400 μM of the total amount, final concentration of limiting substrate inside cells: 15 μM).

Catalyst	FI	Product formation (μM)	Product formation/cell (μM/cell)	Yield
**L3** (50 μM)	6483	0.41	2.39E-06	2.7%
**C5** (50 μM)	14590	2.71	1.58E-05	18%
**C5** (25 μM)	11075	1.57	9.16-06	10.4%

S13.- References

(1) Mummidivarapu, V. V. S.; Hinge, V. K.; Samanta, K.; Yarramala, D. S.; Rao, C. P. *Chem. Eur. J.* **2014**, *20*, 14378.

(2) Hsieh, Y. C.; Chir, J. L.; Wu, H. H.; Chang, P. S.; Wu, A. T. *Carbohydr. Res.* **2009**, *344*, 2236.

(3) Del Amo, D. S.; Wang, W. *J. Am. Chem. Soc.* **2010**, *132*, 16893.

(4) Besanceney-Webler, C.; Jiang, H.; Zheng, T.; Feng, L.; Del Amo, D. S.; Wang, W.; Klivansky, L. M.; Marlow, F. L.; Liu, Y.; Wu, P. *Angew. Chem. Int. Ed.* **2011**, *50*, 8051.

(5) Tomás-Gamasa, M.; Martínez-Calvo, M.; Couceiro, J. R.; Mascareñas, J. L. *Nat. Commun.* **2016**, *7*, 1.

(6) Lal, S.; McNally, J.; White, A. J. P.; Díez-González, S. *Organometallics* **2011**, *30*, 6225.

(7) Díez-González, S.; Nolan, S. P. *Angew. Chem. Int. Ed.* **2008**, *47*, 8881.

(8) Haldón, E.; Delgado-Rebollo, M.; Prieto, A.; Álvarez, Maya, C.; Nicasio, M. C.; Pérez, P. J. *Inorg. Chem.* **2014**, *53*, 4192.
